# Whole cell inactivated poly-bacterial preparation MV130 effect on nasal mucosal immunity and experimental human pneumococcal carriage: double-blind randomised controlled trial with controlled human infection model

**DOI:** 10.1016/j.ebiom.2026.106443

**Published:** 2026-08-26

**Authors:** John Ndaferankhande, Kondwani C. Jambo, Tarsizio Chikaonda, Godwin Tembo, Laura Conejero, Marc Y.R. Henrion, Evaristar Kudowa, Gareth Lipunga, Anthony E. Chirwa, Edna Nsomba, Mphatso C. Mayuni, Mphatso Mailboy, Andrew Sigoloti, Brenald Dzonzi, Edward Mangani, Vitumbiko Nkhoma, Shalom Songolo, Neema Toto, Faith Thole, N. Peter K. Banda, Bridgette Galafa, Glory Kadzanja, Morrison P. Kamanga, Lorensio Chimgoneko, Tiyamike Nthandira, Benson M. Tembo, Alfred Muyaya, Lumbani Makhaza, Bright Lipenga, Vusumuzi Katangwe, Gift Chiwala, Jonathan Grigg, Stephen B. Gordon, MARVELS consortium, Mark Alderson, Sandra Antoine, Christopher R. Bailey, Debbie Bogaert, Jeremy Brown, Sarah Burr, Marien L. De Jonge, Klara Doherty, David Goldblatt, Gabriela Gomes, Joel Gondwe, Kate Gooding, Tina Harawa, Rob Heyderman, Jason Hinds, Simon Jochems, Blessings Kapumba, Vella Kaudzu, Robert Kneller, Richard Malley, Jane Mallewa, Lucinda Manda-Taylor, Henry Mwandumba, Percy Mwenechanya, Mike Parker, Andrew Pollard, Modesta Reuben, Jeffrey Weiser

**Affiliations:** a Malawi-Liverpool Wellcome Research Programme, Blantyre, Malawi; b Department of Clinical Sciences, Liverpool School of Tropical Medicine, Pembroke Place, Liverpool, L3 5QA, UK; c Inmunotek, S.L. Alcalá de Henares, Spain; d Department of Medicine, Kamuzu University of Health Sciences, Queen Elizabeth Central Hospital, Blantyre, Malawi; e Blizard Institute, Queen Mary University of London, London, UK; f Centre for Inflammation Research, Institute for Regeneration and Repair, University of Edinburgho, Little France, Edinburgh, UK

**Keywords:** Trained immunity, Vaccine, CHIM, Mucosal immunity, *Streptococcus pneumoniae*

## Abstract

**Background:**

Bacterial mucosal immunotherapy has shown protection of children and adults from both viral and bacterial respiratory infections, offering the potential to reduce antimicrobial use, and hence also control antimicrobial resistance (AMR). Pneumococcal carriage of vaccine type *Streptococcus pneumoniae* remains high in Malawi despite infant conjugate vaccination and AMR is increasing. We compared nasal inflammation following sublingual bacterial immunotherapy including *S. pneumoniae* (MV130, Inmunotek, Spain) or placebo and determined the effect in an experimental human pneumococcal carriage model.

**Methods:**

A double-blind, randomised, placebo-controlled trial in healthy adult volunteers was conducted at Queen Elizabeth Central Hospital in Blantyre, Malawi. Participants were randomly allocated to receive MV130 or placebo sublingually once daily for 42 days. Mucosal inflammation (neutrophil to T cell ratio, NTR) was measured in nasal micro-biopsies. Post-treatment, participants were challenged with 160,000 CFU/naris *S. pneumoniae* 6B (Spn6b). Experimental pneumococcal carriage rates post inoculation were compared between the two arms. All participants completing the study were included in the analysis. Prospective trial registration: PACTR202403820001276.

**Findings:**

107 participants were enrolled and randomised to MV130/placebo between May and December 2024. There were no serious adverse events, complete compliance was good (72%) and all adverse events were mild. 96 participants (53 male, 43 female) completed the study with 52 participants randomised to MV130 and 44 to placebo. There was no difference in mucosal inflammation (neutrophil to T cell ratio) at day 14 of the intervention MV130 NTR median = 0.737 (IQR 0.294, 2.059) and placebo NTR = 0.831 (IQR 0.450, 2.073), p = 0.64. Secondary analyses showed a rise in mucosal neutrophils after MV130 treatment and after experimental pneumococcal inoculation. There was no difference in nasal or serum anti-pneumococcal immunoglobulin or in experimental pneumococcal carriage proportion between MV130 (12/52, 23%) and placebo (10/44, 23%) groups (unadjusted risk ratio 1.02 (CI 0.49–2.12) p = 1.0).

**Interpretation:**

MV130 induced non-specific mild neutrophil inflammation of the nasal mucosa but had no protective effect against experimental human pneumococcal carriage.

**Funding:**

Wellcome Trust.

**Research in context**

**Evidence before this study**

Respiratory tract infections and antimicrobial resistance remain major public health challenges in low-income countries such as Malawi. Despite the introduction of pneumococcal conjugate vaccines, *Streptococcus pneumoniae* continues to circulate through persistent carriage, serotype replacement, and ongoing transmission, contributing to the emergence of antimicrobial-resistant strains. In this context, non-antibiotic strategies capable of modulating mucosal immunity and reducing pneumococcal carriage warrant investigation.

We searched PubMed for articles published in the 10 years up to February 2026, using the terms (“Polyvalent bacterial preparation” OR “immunostimulation” OR “bacterial lysates” OR “MV130” OR “OM-85″) AND (“respiratory infection”) which identified 18 articles. When the search was refined to include “immunomodulation” AND “*S. pneumoniae*” OR “carriage”, no studies in humans were retrieved.

MV130 is a trained immunity-based mucosal vaccine with immunomodulatory properties that has been shown to prevent respiratory tract infections across multiple clinical settings. A Phase 3 clinical trial of MV130 immunotherapy showed reduction in recurrent wheezing in a cohort of 120 children. MV130 was shown to reduce experimental viral infections, and a different product (polyvalent mechanical bacterial lysate, PMBL) has been shown to eliminate *Staphylococcus aureus* carriage in children treated for grass pollen allergy. Animal studies have shown that a lysate of non-typable *Haemophilus influenzae* delivered as an inhalation to mice protected the mice from a lethal inhalation dose of *S. pneumoniae* serotype 4. More recently, trained immunity (virally induced) has been shown to improve survival from pneumococcal infection of the lung by induction of memory alveolar macrophages. In separate work, bacterial lysates prepared by both alkaline and mechanical disruption and delivered intranasally were shown to offer protection against pneumococcal infection (serotype 1) in mice independent of neutrophils, IL-17A or caspase-1 activation.

**Added value of this study**

This randomised clinical trial evaluating MV130, a mucosal bacterial vaccine, demonstrated a mucosal immunomodulatory effect of MV130 in a Double Blinded Randomised Controlled Trial (DBRCT), with no impact on experimental human pneumococcal carriage.

**Implications of all the available evidence**

Previous clinical studies have demonstrated protection of MV130 against recurrent respiratory tract infections of different aetiology, including children with recurrent wheezing. MV130 did not result in immunity against pneumococcal carriage in this experimental human pneumococcal carriage model. Further clinical and experimental studies are required to understand whether MV130 can protect against natural pneumococcal infections or co-infections.

## Introduction

Bacterial mucosal immunotherapy, using bacterial extracts or whole-cell inactivated bacteria, has mucosal immunity-modulating functions that have the potential to reduce respiratory tract infections in an antigen-independent manner.^1^ The protective effects of bacterial immunotherapy have been shown to extend to both viral^2^ and bacterial infections.^3^ Although the mechanisms of protection are not fully defined, putative mechanisms include dendritic cell stimulation,^4^ epigenetic modification^5^ and increased cytokine production upon a second restimulation,^4^^,^^6^ collectively termed trained immunity.^1^ In placebo controlled randomised controlled trials (RCTs) in patient populations overall results are variable with the European Medicines Agency concluding that there is some evidence of effectiveness of bacterial extracts in the prevention of recurrent respiratory tract infections.^7^ A systematic review of the bacterial lysate OM-85 reported a reduction in total acute respiratory tract infections (ARTI) in children.^8^ Since many of these viral ARTIs are treated empirically with antibiotics, the resulting indiscriminate use of antibiotics contributes to antimicrobial resistance (AMR). Trained immunity and its potential heterologous protection have led to the concept of Trained Immunity based Vaccines (TIbV), which represent a novel approach in vaccinology.

Respiratory tract infections and AMR are major challenges in Malawi. *Streptococcus pneumoniae* remains a significant pathogen despite the roll-out of pneumococcal conjugate vaccination (PCV), owing to pressure of infection, ongoing vaccine-type carriage and transmission, serotype replacement and non-vaccine type colonisation.^9^ This persistence of pneumococcal carriage has been associated with an increase in anti-microbial resistant strains of pneumococci.^10^

We have developed an Experimental Human Pneumococcal Challenge (EHPC) model in which healthy volunteers are inoculated with *S. pneumoniae* type 6B (Spn6b). This controlled human infection model (CHIM)^11^ has been used to successfully confirm PCV13 protective efficacy in both UK^12^ and Malawian^13^ studies and has also been useful in identifying correlates of protection from pneumococcal carriage in healthy^14^ and vulnerable populations. The EHPC model is applicable for testing co-infection effects with viruses^15^ and other pneumococcal types acquired by concurrent natural infection.^16^ In this study we sought to use our model to assess the effect of pre-treatment with a bacterial immunotherapy. We chose to use a whole cell inactivated poly bacterial preparation MV130. MV130 consists of six different bacterial strains in different proportions namely: *S. pneumoniae* (60%), *Staphylococcus aureus* (15%), *Staphylococcus epidermidis* (15%), *Klebsiella pneumoniae* (4%), *Moraxella catarrhalis* (3%) and *Haemophilus influenzae* (3%).^2^ These bacterial isolates are often found in the human upper respiratory tract and MV130 is currently available in some EU countries as a named-patient product.

A lysate of non-typable *H. influenzae* delivered as an inhalation to mice protected the mice from a lethal inhalation dose of *S. pneumoniae* serotype 4.^17^ More recently, trained immunity (virally induced) has been shown to improve survival from pneumococcal infection of the lung by induction of memory alveolar macrophages.^18^ In separate work, bacterial lysates prepared by both alkaline and mechanical disruption and delivered intranasally were shown to offer protection against pneumococcal infection (serotype 1) in mice independent of neutrophils, IL-17A or caspase-1 activation.^19^

Given the persistence of pneumococcal carriage of vaccine-type pneumococci in Malawi following PCV roll-out, the ongoing risk of transmission and infection, and the additional threat of increasing AMR, we wished to determine in an exploratory study if a mucosal bacterial immunotherapy might modulate mucosal immune response and have an impact on pneumococcal carriage in our population. We recruited healthy adult volunteers to a DBRCT in which sublingual MV130 treatment for 42 days was compared to placebo. We aimed to determine acceptability, safety, and mucosal immunomodulatory effects of MV130 in healthy Malawian adults, and as an exploratory secondary endpoint, to assess the effect of MV130, which includes *S. pneumoniae* serotype 6B as one of 9 serotypes represented, on nasal colonisation in a controlled human infection model of pneumococcal carriage.

## Methods

### Study design, location

The study was a double-blind, randomised, placebo-controlled trial (DBRCT) conducted at the Queen Elizabeth Central Hospital (QECH) in Blantyre, Malawi. Recruitment and sample collection from participants took place at the Malawi Accelerated Research by Vaccine and Experimental Laboratory Systems (MARVELS) clinic at QECH as illustrated in Supplementary Figure S1B. Study product dispensing and experimental inoculation with Spn6b took place in the same clinic. Laboratory analysis for immunological end points, and microbiology culture and PCR was performed at the Malawi Liverpool Wellcome Programme laboratory.

### Ethics

The study design was informed by the MARVELS programme of public engagement events and focus groups. Consistent with the Declaration of Helsinki, the protocol was approved by the National Health Science Research Ethics Committee (NHSRC Reference AD23/04/4041) in Malawi and in the UK by the Liverpool School of Tropical Medicine Research Ethics Committee (23-017). The study was also approved by the Pharmacy & Medicines Regulatory Authority of Malawi (registration number PMRA/CTRC/III/07082023154) and monitored by a Data and Safety Monitoring Board (DSMB) constituted for the trial. The trial was registered on 28/03/24 with the Pan African Clinical Trial Registry (identification number PACTR202403820001276). https://pactr.samrc.ac.za/All participants gave written informed consent, which included a comprehension assessment.

### Participant screening

Healthy Malawian adult volunteers aged 18–40 years with no history of pneumococcal disease or vaccination were recruited. Eligibility was confirmed through medical history including self-reported sex, physical examination, and blood screening tests, including HIV testing and pregnancy testing. Immunocompromised participants or those with social contacts deemed at risk of invasive pneumococcal disease (e.g. elderly or infants) were excluded in line with our safety procedures developed in previous experimental pneumococcal carriage studies.^13^ Full inclusion and exclusion criteria have been published in the study protocol (https://wellcomeopenresearch.org/articles/10-479).

### Randomisation and masking

#### Randomisation

The study statistician (MH) used a random number generator to generate the randomisation sequence and randomisation occurred using block randomisation with random block sizes of 4, 6, and 8 to ensure balanced product allocations. An independent statistician ran the randomisation code for study group allocations. Allocations were stored on a secure electronic portal at the Malawi Liverpool Wellcome Programme (MLW).

#### Blinding and intervention

Participants were individually randomised 1:1 to MV130 or placebo before the first dose administration of the study product. The pharmacy team was fully unblinded and received the numbered treatment according to the randomisation code. Identically packaged individual MV130/placebo kits were prepared to maintain research team and participant blinding. Individual MV130/placebo kits were identified only by a randomisation identification number. Unblinding of the entire study team (clinic, labs, data, statistics) only occurred once microbiological reporting was completed for the final participant at the final visit.

**MV130/placebo** were given sublingually for 42 days. Both MV130 and placebo were manufactured and supplied by Inmunotek, S.L. Spain. MV130 formulation contains as active ingredient/s different species of whole-cell inactivated bacteria at 300 formazin turbidity units which are frequently present in the respiratory tract: V102 *S. aureus* (15%), V101 *S. epidermidis (*15%), V104 *S. pneumoniae (*60%), V113 *K. pneumoniae (*4%), V105 *M. catarrhalis (*3%) and V103 *H. influenzae (*3%). The placebo included only the excipients which were glycerol, pineapple artificial flavouring, sodium chloride and water for injection. The products were administered to the sublingual mucosa daily via two sprays from a metred pump. Each spray was 0.1 ml leading to a daily dose of 0.2 ml. The first dose was administered at the clinic under the supervision of clinical trial staff, and the remaining doses were administered by the participants themselves, or by direct observation at clinic. Six-week exposure (42 days) was chosen as sufficient to elicit an effect without burdening participants with a long period of compliance.

### Clinical study procedures

#### Study samples

Enrolled participants allowed collection of baseline nasal samples including nasosorption fluid by filter paper adsorption and nasal scrape for mucosal immunological assessment and nasal wash for determination of pneumococcal carriage. Whole blood and serum were collected for humoral and cellular immunological analyses. Nasal and blood samples matched to the baseline samples were collected after 14 and 35 days of MV130/placebo administration, and 2 days and 7 days after pneumococcal inoculation.

### Outcome measurements

#### Endpoint assessment—immunology

Nasal cell samples collected as nasal scrapes were transported to the laboratory in complete media using cold boxes with melting ice. These samples were processed immediately as follows. Briefly, cells were dislodged from the nasal scrapes by pipetting 5 times up and down. The suspension was spun at 500 g for 8 min at 4 °C and re-suspended with an appropriate dilution of viability stain (Live-dead APC) to exclude the dead cells and incubated in the dark for 15 min on ice. An antibody cocktail was prepared with a panel containing markers for epithelial cells, lymphocyte subsets and activation and neutrophils (EPCAM BV605, CD3 APC-CY7, CD45 PerCP5.5, CD66b FITC). Antibody details in Supplementary Table S2. An appropriate dilution of antibody cocktail was then added and incubated on ice for 10 min. Samples were washed in 1 ml of phosphate buffer saline (PBS) before re-suspension in PBS and acquisition on a BD LSR Fortessa flow cytometer.

The primary endpoint of neutrophil to T-cell ratio (NTR) was chosen because neutrophils are recruited to the nasal mucosa and are active in opsonophagocytic killing of *S. pneumoniae*. The NTR was determined using a flow cytometry gating strategy described in Supplementary Figure S1A.

#### Pneumococcal anti-capsular polysaccharide IgG antibody ELISA

Pneumococcal anti-capsular polysaccharide antibody ELISA: We measured the concentration of IgG antibodies to the capsular polysaccharide of Spn6b in sera and nasal lining fluid using the World Health Organisation enzyme-linked immunosorbent assay (ELISA) protocol for the detection of anti-pneumococcal capsular polysaccharide antibodies. In brief, ELISA plates were coated with 5 μg/ml of serotype-6B capsular polysaccharide and incubated at 2–8 °C overnight (9–15 h). Plates were washed three times with PBS-Tween 20 (Sigma) before adding samples or Human Anti-Pneumococcal capsule Reference Serum 007sp (National Institute for Biological Standards and Control) diluted in 5 ng/μl CWPS Multi (SSI Diagnostica). Four and seven 2.5-fold serial dilutions were performed for samples and standards, respectively and added to the ELISA plate in triplicate before incubation at room temperature for 2 h. Plates were washed 5 times with 0.05% PBS-Tween 20 before adding Goat anti-Human IgG-Alkaline Phosphatase (Southern Biotech) and incubated in the dark at room temperature for 2 h. After washing 5 times with PBS Tween 20, we added p-nitrophenyl phosphate substrate (Sigma) and incubated the plates at room temperature in the dark for 30 min. We measured optical densities at 405 nm and calculated concentrations using a four-parameter logistic curve in MyAssays online platform. We generated a standard curve for each ELISA plate.

#### Determination of pneumococcal carriage

After 42 days of sublingual MV130 or placebo administration, all participants were experimentally inoculated with *S. pneumoniae* 6B (Spn6b) strain BHN418 (GenBank https://www.ncbi.nlm.nih.gov/nuccore/ASHP00000000.1) at a dose of 160,000 colony forming units (cfu) in each naris. Inoculation was carried out after the full treatment period and safety screening, also allowing any inflammation from nasal sampling to resolve. Nasal wash samples were collected on day 2 and day 7, following *S. pneumoniae* inoculation, as previously described, and the nasal wash pellet plated for culture as previously described.^20^ Briefly, nasal wash samples were centrifuged at 3400×*g* for 10 min upon receipt in the lab. Following centrifugation, supernatant was stored at −80 °C. The nasal wash bacterial pellet was resuspended in 100 μl of skim milk, tryptone, glucose, and glycerol (STGG). Eight 1:10 serial dilutions were prepared with 20 μl of the STGG nasal wash pellet and plated onto Columbia blood agar (Oxoid, Basingstoke, UK) supplemented with 5 μg/ml gentamicin, to establish the number of Colony Forming Units (CFUs). Plates were incubated for 18–24 h at 37 °C in 5% CO_2_ and examined for colonies displaying typical pneumococcal morphology. The remaining STGG suspended pellet was stored for molecular analysis.

We distinguished experimental human pneumococcal carriage (EHPC) with the inoculated Spn6b strain from natural carriage (NC) resulting from incidental pneumococcal colonisation during the study by serotyping cultured pneumococci sampled from plates. Nasal wash plate colonies confirmed as *S. pneumoniae* using classical microbiological techniques were typed by latex agglutination using Immulex Pneumotest reagents (Statens Serum Institut, Copenhagen, Denmark) to confirm pneumococcal serogroup. EHPC was defined as detection of Spn6b in either of the nasal washes taken at 2 and at 7 days post inoculation.

Stored STGG pellets were examined with a dual quantitative PCR (*lytA* and *cpsA*) used to confirm EHPC and distinguish this from NC. Briefly, genomic bacterial DNA was extracted from STGG nasal wash pellet samples described above using the Agowa Mag Mini DNA Extraction Kit (LGC Genomics, Berlin, Germany) according to the manufacturer's instructions. The extracted DNA was stored at −20 °C for subsequent *cps*A/*lyt*A multiplex quantitative PCR (qPCR). The primer and probe sequences used for qPCR were as follows: *cps*A forward primer 5ʹ-AAGTTTGCACTAGAGTATGGGAAGGT-3ʹ; *cps*A reverse primer 5ʹ-ACATTATGTCCATGTCTTCGATACAAG-3ʹ; *cps*A probe 5ʹ-TGTTCTGCCCTGAGCAACTGG-3ʹ and *lyt*A forward primer 5ʹ-ACGCAATCTAGCAGATGAAGCA-3ʹ; *lyt*A reverse primer 5ʹ-TCGTGCGTTTTAATTCCAGCT-3ʹ; *lyt*A probe 5ʹ-TGCCGAAAACGCTTGATACAGGGAG-3ʹ. Each 20 μl reaction mixture contained 1 μl of each primer, 0.4 μl of each probe, 2.7 μl of PCR-grade water, 10 μl of qScript XLT 1-Step RT-qPCR ToughMix (Quantabio, Cummings Centre, Beverly, MA, USA), and 2.5 μl of DNA sample, prepared in duplicate wells. Quantitative PCR was performed on a QuantStudio 7 Flex instrument (Applied Biosystems, Lincoln Centre Drive, Foster City, CA, USA) under the following cycling conditions: 95 °C for 10 min, followed by 40 cycles of 95 °C for 15 s and 60 °C for 1 min. PCR was determined by a copy number less than that of the lowest dilution in the standard curve for both *lytA and cpsA*.

We estimated pneumococcal carriage density for experimental and natural carriage by a colony count of the plated nasal wash and for Spn6b only by the copy number of the PCR.

#### Treatment and recording of adverse events

To assess safety, all Adverse Events (AEs) occurring during the trial, from the time of signature of the informed consent, to the completion of the trial, were recorded and evaluated. Unsolicited events were recorded in participant diaries and solicited events were recorded during clinic visits.

#### Measures of compliance

Daily records of treatment compliance were kept by all participants, and the dispensed product (MV130/placebo) was weighed before dispensing and on return to the research clinic to determine used dose. Self-reported compliance was reported as complete if treatment was taken on 38 or more of the 42 days of treatment which corresponds to 90% compliance. Full bottles weighed 39g and 10g of this weight corresponded to 84 sprays. Participants with bottle weights of less than 30g were therefore deemed 90% compliant. Overall compliance was determined as being both self-reported and bottle weight compliant.

### Statistics

The pre-specified primary end point was chosen as the neutrophil to T cell ratio in nasal mucosa at day 14 of treatment as this was the earliest time point at which we expected to see a difference between MV130 and placebo. The pre-specified primary efficacy analysis compared log (NTR) between participants randomised to MV130 and controls using a non-parametric Wilcoxon rank-sum test. To inform the sample size calculations, the distribution of NTR and log (NTR) measurements observed in a previous EHPC study were used. The standard deviation for log (NTR) was estimated as σ = 1.2 and therefore with 100 participants, the study would be able to detect a difference in log (NTR) between both groups of 0.69 with 80% power. This corresponded to a standardised effect size of 0.57 which is typically regarded as a moderate effect size. Pre-specified secondary analyses included comparisons of NTR at other timepoints, comparisons between timepoints and day 0, and analyses adjusted for individual participant characteristics that could impact the endpoint including study product compliance.

The pre-specified secondary endpoint was the nasal pneumococcal carriage rates at days 2 and 7 post-inoculation. Further analyses associated the study allocation and pneumococcal carriage density (pre-specified), as well as the effect of MV130 on the incidence of natural carriage (not pre-specified). The trial is reported in accordance with CONSORT 2025 guidelines. The statistical analysis plan, which lists all pre-specified analyses, is available from GitHub (https://github.com/mlw-stats/MARVELS_MV130). This repository also includes the full analysis code.

### Role of funders

The work leading to the results published in this article received funding from the Wellcome Trust through the Malawi Accelerated Research on Vaccines by Experimental Systems Grant (grant 211433/Z/18/Z) to SBG. The MV130 and placebo were provided by Inmunotek, Spain. The funder had no role in the study design, data collection and analysis, decision to publish, or preparation of the manuscript.

## Results

### Recruitment

**Fig. 1:**
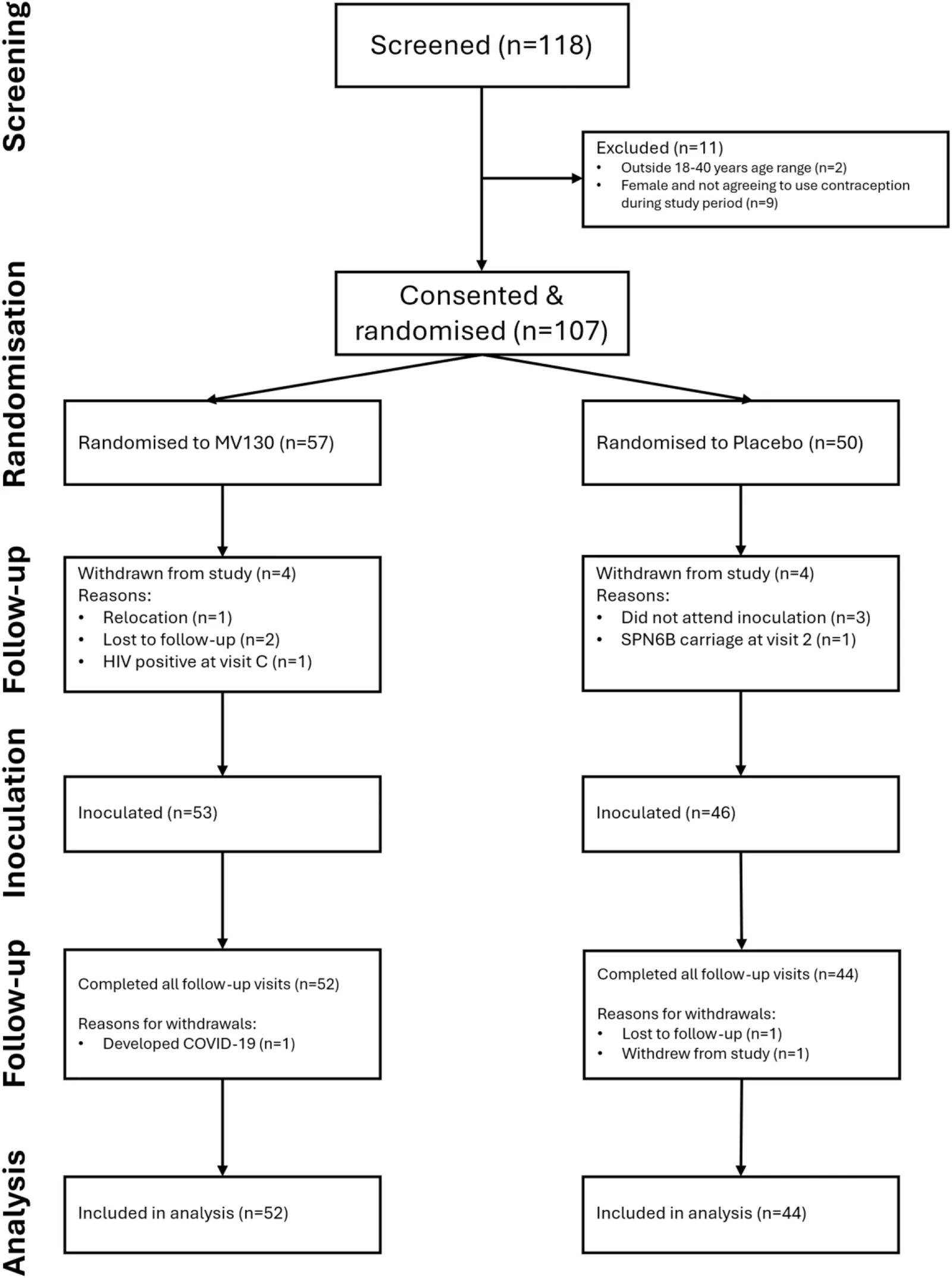
Consort diagram.

Recruitment started on 21st May 2024, and the last participant follow-up visit was completed on 24th December 2024. 118 volunteers were screened and 107 proceeded to randomisation (57 to MV130, 50 to placebo) as shown in Fig. 1. Full data and samples were collected for 96 subjects (52 in MV130 group, 44 in placebo). The participant demographics are shown in Table 1.

**Table 1:** Participants’ demographic characteristics at screening.

Study characteristics	All	MV-130	Placebo
Participants n (%)	96 (100.0%)	52 (54.2%)	44 (45.8%)
Sex: male n (%)	53 (55.2%)	25 (48.1%)	28 (63.6%)
Age median (IQR)	26.85 (23.75, 30.12)	26.86 (23.42, 29.75)	26.75 (23.98, 31.14)
BMI median (IQR)	22.53 (20.76, 25.06)	23.27 (20.67, 25.27)	22.20 (20.81, 24.83)
Smoking status: never smoker n (%)	94 (97.9%)	51 (98.1%)	43 (97.7%)
Smoking status: past smoker n (%)	0 (0.0%)	0 (0.0%)	0 (0.0%)
Smoking status: current smoker n (%)	2 (2.1%)	1 (1.9%)	1 (2.3%)

### Adherence to treatment and adverse events

Self-reported adherence to treatment was very high, with 91/96 (95%) of participants compliant. Bottle weight showed 71/96 (74%) compliance, with 69/96 (72%) compliance by both methods. No participant dispensed less than half of the medication by weight (Supplementary Figure S6). By allocation, 33/52 (63%) participants in the MV130 group and 36/44 (82%) in the placebo participants group were compliant by both methods. Enquiry revealed that difficulties in elevating the tongue were a challenge for some participants (fraenulum restriction). We included all participants in an Intention to Treat (ITT) analysis as per the published protocol.

There were no Serious Adverse Events (SAE) in this study, and Adverse Events (AEs) were very uncommon. In total, 20 participants experienced an AE, 12 from the placebo arm and 8 from the MV130 arm. All AEs were mild, with 10 participants presenting unsolicited AE, and a further 10 reporting mild symptoms only when asked. None of the AE reported were related to the medication. Full details are presented in the Supplementary Figure S2.

### Primary endpoint neutrophil to T cell ratio

**Fig. 2:**
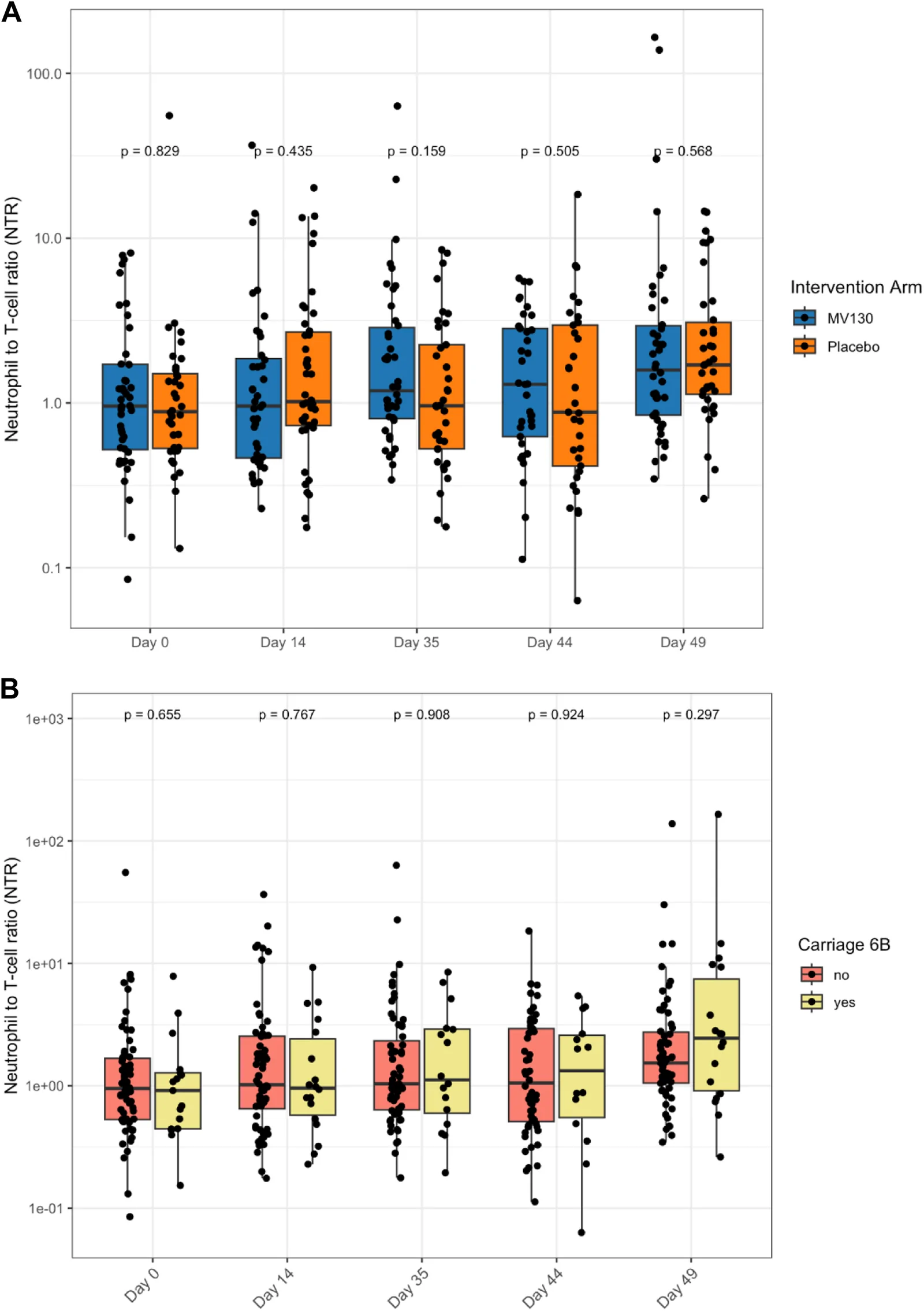
Neutrophil to T cell ratio (NTR) measured by flow cytometry using nasal mucosal scrape samples. Upper panel (A) NTR data shown by treatment status at baseline, day 14, and day 35 of treatment as well as 2 and 7 days after inoculation (day 44 and day 49). Lower panel (B) NTR data compared by experimental carriage status and timepoint. The middle bar in each box represents the median with the lower and upper boundary of the box representing the 25th and 75th percentiles respectively.

The neutrophil to T cell ratio (NTR) in nasal scrapes analysed by flow cytometry showed a non-normal distribution even after log transformation, and data were therefore compared using the Wilcoxon rank-sum test. The pre-specified primary endpoint data showed no difference in baseline or day 14 NTR between the MV130 treatment group and placebo, and this was the case at all other study visits, as shown in Fig. 2 (pre-specified between-group comparisons of day 0 to day 49 shown in Fig. 2, and by sex in Supplementary Figure S7). However, in post-hoc within-group analyses, there was a rise (compared to the baseline visit) in NTR in the MV130 group, but not in the placebo group, at the Day 35 visit. There was also a rise in both MV130 and placebo groups 7 days (Day 49 in the study schedule) after inoculation as shown in Supplementary Table S1. There was no difference in the primary endpoint of NTR at day 14 between compliant and non-compliant participants as shown in Supplementary Figure S3, nor was there a difference in carriage rates (secondary endpoint) between study arms when only compliant participants were analysed (Supplementary Table S3).

### Immunoglobulin response in nasal lining fluid and serum

**Fig. 3:**
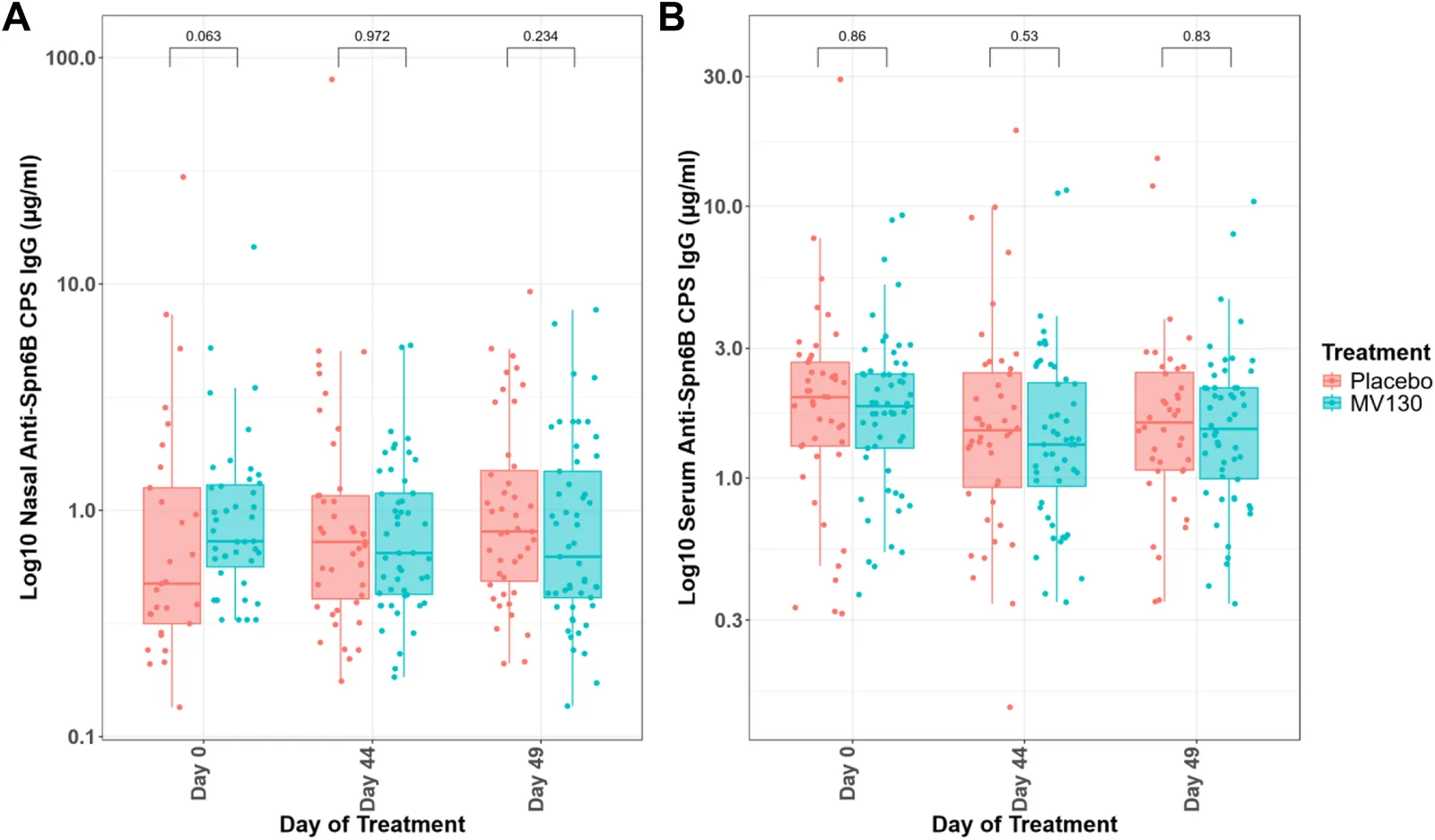
Concentration of binding anti-Spn6B CPS IgG in nasal lining fluid (Panel A) and serum (Panel B) of MV130 and placebo recipients before the start of treatment (Day 0), Day 44 after treatment initiation (day 2 post-experimental Spn6B inoculation), and Day 49 after treatment initiation (day 7 post-inoculation). At Day 0, nasal anti-Spn6B CPS IgG geometric mean concentrations (GMCs) were 0.72 μg/mL (95% CI 0.45–1.14 μg/mL) in the placebo group and 0.90 μg/mL (95% CI 0.70–1.14 μg/mL) in the MV130 group. At Day 44, nasal GMCs were 0.86 μg/mL (95% CI 0.61–1.22 μg/mL) and 0.76 μg/mL (95% CI 0.61–0.94 μg/mL) in the placebo and MV130 groups respectively. At Day 49, nasal GMCs were 0.96 μg/mL (95% CI 0.72–1.28 μg/mL) in the placebo group and 0.77 μg/mL (95% CI 0.60–1.00 μg/mL) in the MV130 group. In serum Day 0 anti-Spn6B CPS (GMCs) were 1.76 μg/mL (95% CI 1.36–2.27 μg/mL) in the placebo group and 1.74 μg/mL (95% CI 1.46–2.09 μg/mL) in the MV130 group. At Day 44, GMCs were 1.53 μg/mL (95% CI 1.16–2.01 μg/mL) in the placebo group and 1.39 μg/mL (95% CI 1.13–1.71 μg/ml) in the MV130 group. At Day 49, serum GMCs were 1.59 μg/mL (95% CI 1.25–2.03 μg/mL) in the placebo group and 1.51 μg/mL (95% CI 1.26–1.81 μg/mL) in the MV130 group.

We wished to determine if anti-pneumococcal capsular polysaccharide immunoglobulin was either altered by MV130 treatment, associated with differences in defence against carriage, or altered by the inoculation in the experiment. The secondary endpoint of anti-pneumococcal capsular polysaccharide type 6B IgG was determined in nasal lining fluid and serum at each time point. The results indicate that over 42 days of MV130 treatment, MV130 did not significantly alter either nasal or serum anti-capsular type 6B IgG levels (Fig. 3).

A pre-specified secondary comparison of the association of serum and nasal anti-Spn6bCPS binding antibodies on experimental carriage of Spn6b was made. At baseline the nasal anti-Spn6B CPS IgG levels were higher in participants who later remained carriage negative than in those who later became carriage positive. There were no significant differences in the two groups, however, at the subsequent measured time points (Supplementary Figure S4A). When grouped by treatment arm, no significant differences in nasal IgG were observed between carriage-negative and carriage-positive participants in the placebo group at any time point (Supplementary Figure S4B). However, in the MV130 group at baseline, nasal IgG geometric mean concentrations (GMC) were higher in carriage-negative individuals than in those who became carriage-positive. This trend continued at day 44, with carriage-negative showing higher IgG levels compared with the carriage-positive group. On day 49, nasal IgG GMC were similar between the two groups (Supplementary Figure S4B). In addition, in serum, anti-Spn6b CPS IgG GMC did not significantly differ between participants who became carriage-negative and those who became carriage-positive, at any timepoint. This was true when treatment groups were combined (Supplementary Figure S4C) and when participants were split based on treatment group (Supplementary Figure S4D).

### Experimental carriage by treatment group

Twenty-two out of 96 participants in the study (23%) experienced experimental pneumococcal carriage at any time point as determined by culture. This pre-specified secondary endpoint showed 12/52 in the MV130 group (23.1%) and 10/44 in the placebo group (22.7%) as shown in Table 2. Experimental carriage risk ratios for the MV130 arms compared to the placebo arm were: Unadjusted: 1.02, 95% CI (0.49, 2.12) and adjusted: 1.15, 95% CI (0.57, 2.33).

**Table 2:** Study endpoints by experimental group.

	All	MV-130	Placebo
Participants n (%)	96 (100.0%)	52 (100.0%)	44 (100.0%)
Experimental Spn6b Carriage			
Serotype 6B carriage (any timepoint) n (%)	22 (22.9%)	12 (23.1%)	10 (22.7%)
Serotype 6B carriage (day 2 post-inoculation) n (%)	18 (18.8%)	9 (17.3%)	9 (20.5%)
Serotype 6B carriage (day 7 post-inoculation) n (%)	13 (13.5%)	8 (15.4%)	5 (11.4%)
Neutrophil to T cell ratio			
NTR (day 0) median (IQR)	0.64 (0.38, 1.81)	0.66 (0.38, 1.98)	0.62 (0.39, 1.48)
NTR (day 14) median (IQR)	0.79 (0.31, 2.07)	0.74 (0.29, 2.06)	0.83 (0.45, 2.07)
NTR (day 35) median (IQR)	1.25 (0.72, 3.92)	1.57 (0.97, 4.08)	0.82 (0.45, 2.48)

**Fig. 4:**
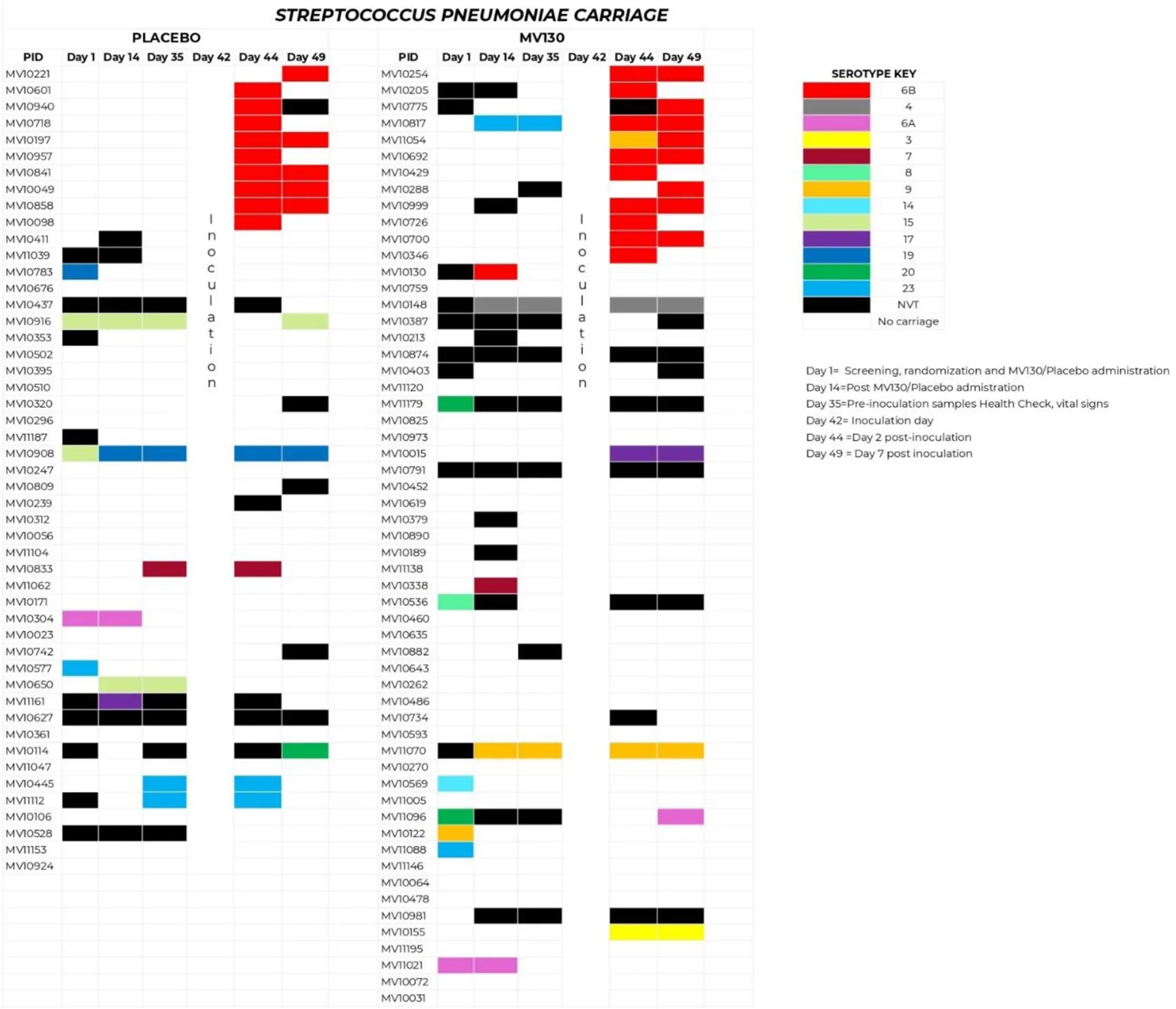
Experimental human pneumococcal carriage (EHPC) and natural carriage (NC) by pneumococcal serotype shown by treatment allocation (MV130 in the left-hand panel, placebo on the right). Inoculation with SPN6B (shown in red) occurred on day 42 and was followed by EHPC in 12/52 MV130 (23%) and 10/44 (23%) in the placebo participants. Natural carriage was observed in similar proportions among MV130 and placebo participants, stratified by serotype according to the key (NVT: non-vaccine type). There was one episode of natural 6b carriage on day 14 in the MV130 group.

Experimental and natural carriage are described by treatment arm and time-point in Fig. 4. There was no statistically significant difference in the measured NTR when participants with experimental carriage were compared with those with no experimental carriage as shown in Fig. 2B. Further, when carriage density was estimated for EHPC using plate dilution and Spn6b dual PCR and for natural carriage using plate dilution alone, there were no differences observed between the treatment arms at any time point as shown in Supplementary Figure S5.

### Natural pneumococcal carriage by treatment status

The incidence of experimental carriage and natural carriage (NC) events in each treatment arm by participant and date is summarised in Fig. 4. There were similar numbers of events and a similar range of serotypes between the two study arms.

## Discussion

We found in a group of healthy adult volunteers that MV130 had no effect on our predefined primary endpoint (nasal mucosal neutrophil to T cell ratio) at day 14 and there was also no effect on the incidence or density of experimental human pneumococcal carriage when volunteers were inoculated after 42 days of treatment, at day 2 and day 7 post inoculation. There was, however, an increase in the nasal mucosal neutrophil to T cell ratio (NTR) measured in participants taking MV130 when comparing day 0 to day 35, that was not found in participants taking placebo.

The strengths of this study include its double-blind, randomised, placebo-controlled design and sufficient statistical power to reject the primary hypothesis of acute mucosal inflammation. Secondary analyses demonstrated neutrophil infiltration of the nasal mucosa after 35 days, like that seen 7 days after bacterial inoculation. The study demonstrates that MV130 does not elicit a pathogen-specific immune response against *S. pneumoniae* under the model conditions, time point and population assessed.

The study has limitations too. Compliance with the intervention was high, but incomplete in some cases as direct observation daily for 6 weeks was not practicable. The optimal dose of MV130 is not known, and there was no difference in the NTR among MV130 treated participants with high and low compliance. Adherence was somewhat lower in the MV130 group than in the placebo group but subgroup analysis using only fully compliant participant data had no effect on the study endpoints and we have shown partial compliance of at least half the medication by weight in all participants. In addition, the study population consisted of healthy adults—by design we excluded populations that have specifically benefitted from MV130 previously including children,^2^ immunocompromised patients,^21^^,^^22^ and patients with chronic lung disease.^23^ In addition, the carriage rate observed in our study was lower than the reported in previous work using the same model,^12^ and so more virulent strains might be tested in future.^24^

We tested the effect of MV130 on experimental pneumococcal carriage and unlike in studies of pneumococcal conjugate vaccine,^12^^,^^13^ there was no reduction in experimental carriage in the MV130 arm. The mucosal defence against pneumococcus is critically dependent on immunoglobulin, and cellular responses including neutrophils.^14^ We have demonstrated by comparison at the beginning and end of the experiment that neither MV130 nor challenge inoculation alone resulted in a change in mucosal pneumococcal Spn6b capsule-specific IgG. MV130 did, however, increase mucosal neutrophils late during exposure, and we were able to show an increase in nasal IgG against Spn6b capsule after carriage beyond the period of inoculation was established. Neutrophil inflammation can either enhance or decrease pneumococcal carriage depending on context and activation^25^ but in our case, no association was seen between neutrophil inflammation and EHPC at any timepoint. This is consistent with published preclinical data^19^ but surprising given the known mechanisms of protection against pneumococcal carriage.^14^ MV130 has shown protective efficacy against viral infections, likely by post-synaptic dendritic cell activation and this pattern of trained immunity has not been associated with protection against pneumococcal carriage.^26^

It is possible, however, that our selected primary endpoint, chosen based on knowledge of the mechanism of clearance of pneumococcal carriage, and on the availability of nasal mucosal tissue in a human volunteer study was too early, or that we missed an inflammatory response elicited by MV130 on dendritic cells and macrophages.^4^ A characteristic feature of pneumococcal pneumonia, however, is the macrophage and epithelium mediated recruitment of neutrophils to the site of infection, where they are essential for limiting pneumococcal load. The mechanism of defence against pneumococcal carriage is only partially understood but includes anti-capsular immunoglobulin in nasal lining fluid.^26^ It is interesting to note that the experimental pneumococcal carriage rate in this study was highest in the MV130 treated subjects with low anti-capsular IgG to Spn6b capsule.

There are several reasons why MV130 might not elicit a robust immunomodulatory effect in Malawian adults even after 40 days of exposure. First, incident rates of natural pneumococcal carriage are very high among adults in Malawi and so regular mucosal exposure of the nasopharynx to pneumococcal capsule and proteins is the norm.^9^ *S. pneumoniae* forms 60% of the content of MV130, and therefore natural carriage may already have produced the immune response that might have occurred in less exposed populations. Second, there are very few asymptomatic viral co-infections recorded in healthy adult volunteers in CHIM studies in Malawi, and little upper respiratory tract infection seasonality.^16^ This is unlike the UK where experimental carriage was found in 75% of participants with asymptomatic viral infection compared to only 46% with no viral co-infection.^27^ As the prime effect of MV130 may be against viral infection,^6^ with a secondary effect against bacteria, the background inflammation of the nasal mucosa may be very different in the Malawian population. This hypothesis could be tested by using the live attenuated influenza virus and Spn6b co-infection model to determine an effect of MV130 on concurrent viral and bacterial infection.^28^ A further reason for lack of response may be the known regulatory effect of oropharyngeal mucosa cells on host response to bacterial antigens.^29^ It is necessary for the host to co-exist peacefully with nasopharyngeal and oral commensals and as such MV130 and similar bacterial immunotherapies face considerable anti-inflammatory regulation.^30^

Further studies would help to define the effect of MV130 on mucosal or systemic bacterial defence. First, a formal assay of trained immunity might determine if any modulation of the innate response to pathogen signals (LPS and LTA) occurred in the context of MV130 therapy, in this regard, a phase I/II study is currently performed in healthy volunteers in Europe. Secondly, the re-setting of Th1/Th2 balance that has been observed in preclinical work can be sought—the soil helminth exposure common in equatorial regions has previously been thought to drive airway allergen responses but this has been highly context specific. In the absence of symptoms of allergy, it may be that no TIbV effect will be seen in this setting.

We have shown in this study that MV130 did not have a protective effect against experimental pneumococcal carriage but we have not yet examined the effect of MV130 on the wider microbiome, or on trained immunity and these may be useful avenues of further investigation.

## Contributors

Conceptualisation: JG, SBG, JN.

Data Curation: AM, LM.

Formal Analyses including accessing and verifying the underlying data: EK, MH, SBG, JG, VK.

Funding acquisition: SBG, LC.

Investigation: JN, TC, GT, GL, AEC, EN, MCM, EM, VN, MM, AS, SS, NT, FT, NPKB, BG, GK, MPK, LC, TN, BMT, BL, VK, GC, BD.

Methodology: EN, BD, TC, AEC, JN, SBG, KCJ.

Project administration: NT, SBG, BL.

Supervision: KCJ, SBG, JGG.

Writing–original draft: JN, LC, SBG.

Writing–review and editing: all authors.

All authors confirm that they had full access to verify all underlying data in the study and accept responsibility to submit for publication. JG and SBG contributed equally as last authors. All authors read and approved the final version of the manuscript. The Malawi Accelerated Research in Vaccines by Experimental and Laboratory Systems (MARVELS) consortium was formed to establish controlled human infection models in Malawi. MARVELS consortium members beyond those named in the author list contributed to the manuscript by involvement in establishing the Spn6b experimental human carriage model.

## Data sharing statement

A full anonymised dataset can be obtained by applying to Data Research Support Unit, Malawi Liverpool Wellcome Research Programme (email: sdsm@mlw.mw). The Statistical Analysis Plan and analysis code can be obtained from GitHub (https://github.com/mlw-stats/MARVELS_MV130) or by applying to Marc Henrion, Data Science and Computational Modelling Unit, Malawi Liverpool Wellcome Research Programme (mhenrion@mlw.mw).

## Declaration of interests

LC is an employee of Inmunotek, Spain. JG declares unrestricted grants from Inmunotek and OM Pharma, as well as payment to institution from AstraZeneca. All other authors confirm that they have no conflict of interest.

## Appendix A Supplementary data

Supplementary Figures and Tables

Study Protocol

MV130 protocol

Consortium Members
