# Supplementary material for: Whole cell inactivated poly-bacterial preparation MV130 effect on nasal mucosal immunity and experimental human pneumococcal carriage: double-blind randomised controlled trial with controlled human infection model
Source: eBioMedicine. 2026 Aug 26;131:106443. doi: 10.1016/j.ebiom.2026.106443 (PMC13544312; doi:10.1016/j.ebiom.2026.106443)
Supplement: Supplementary file 2 [file mmc1.docx]

Figure S1 Study methods

Figure S1 Panel A Flow cytometry gating strategy for neutrophil to T cell ratio (NTR). Representative gating strategy demonstrating the determination of the neutrophil to T cell ratio. First panel defines singlets, second panel selects leucocytes negative and positive with CD45, side-scatter high cells and counting beads, the third panel selects live cells, the right-hand panel distinguishes neutrophils as CD66b+ cells and lymphocytes as CD3+ cells, and the last panel defines epithelial cells as EpCAM.  The NTR is the number of events in the neutrophil gate divided by the number of events in the lymphocyte gate.

Panel A

Figure Panel B Sample strategy

Figure S2. Participant recorded symptoms (adverse events) by treatment allocation. Unsolicited adverse events were recorded using treatment adherence logs, which were given to participants on the day of treatment commencement, for self-recording of any AEs experienced during course of the study. Solicited adverse events were recorded during the participant visit days at the study clinic. The participants were presented with a set of standardized questions by the study clinic team on both scheduled and unscheduled visits. Adverse events were captured by the clinic team on each visit, and responses were entered on the electronic Case Report Forms.

| **All Adverse Events** | MV130 | Placebo | Total |
| --- | --- | --- | --- |
| joint pain |  | 1 | 1 |
| coryzal symptoms |  | 1 | 1 |
| Cough | 1 | 1 | 2 |
| Dizziness | 2 | 1 | 3 |
| Dry throat at night | 1 |  | 1 |
| Forehead sweating at rest |  | 1 | 1 |
| Headache | 3 | 6 | 9 |
| Headache and stomach-ache |  | 1 | 1 |
| Mild dizziness and numbness | 1 |  | 1 |
|  | 8 | 12 | 20 |

| **Unsolicited adverse events** | MV130 | Placebo | Total |
| --- | --- | --- | --- |
| headache |  | 4 | 4 |
| Dizziness | 2 |  | 2 |
| cough | 1 | 1 | 2 |
| Coryzal symptoms |  | 1 | 1 |
| Joint pain |  | 1 | 1 |
|  | 2 | 7 | 10 |

| **Solicited Adverse Events** | MV130 | Placebo | Total |
| --- | --- | --- | --- |
| Headache | 3 | 2 | 5 |
| Mild dizziness and numbness | 1 |  | 1 |
| Dry throat at night | 1 |  | 1 |
| Forehead sweating at rest |  | 1 | 1 |
| Headache and stomach-ache |  | 1 | 1 |
| Dizziness |  | 1 | 1 |
|  | 5 | 5 | 10 |

Figure S3a NTR at 14 (primary endpoint) by compliance status for MV130 randomised participants. P-value at the top of each study visit are comparing NTR between compliance groups using a two-sample Wilcoxon rank-sum test. There were also no differences between the groups at day 0 and day 35.

Fig S3b Neutrophil absolute cell numbers calculated using bead ratios at days 0, 14, 35, 44 and 49 by study arm. P-value at the top of each study visit are comparing levels between study arms using Wilcoxon’s rank-sum test.

Figure S3c: T-cell absolute cell numbers calculated using bead ratios at days 0, 14, 35, 44 and 49 by study arm. P-value at the top of each study visit are comparing levels between study arms using Wilcoxon’s rank-sum test.

Figure S3d: Neutrophil to epithelial cell ratios at days 0, 14, 35, 44 and 49 by study arm. P-value at the top of each study visit are comparing ratios between study arms using Wilcoxon’s rank-sum test.

Figure S3e: T-cell to epithelial cell ratios at days 0, 14, 35, 44 and 49 by study arm. P-value at the top of each study visit are comparing ratios between study arms using Wilcoxon’s rank-sum test.

Figure S4 Association of nasal lining fluid and serum anti-Spn6B CPS-specific binding antibodies with experimental Spn6B carriage

Panel A Nasal anti SPN6B IgG: At Day 0, nasal anti-Spn6B CPS IgG GMCs were higher in carriage-negative individuals (0.92 µg/mL; 95% CI 0.71–1.19 µg/mL) than in those who became carriage-positive (0.62 µg/mL; 95% CI 0.38–1.02 µg/mL). At Day 44, nasal IgG GMCs were similar between the carriage-negative group (0.82 µg/mL; 95% CI 0.67–1.00 µg/mL) and carriage-positive group (0.77 µg/mL; 95% CI 0.45–1.30 µg/mL). Similarly, at Day 49, nasal IgG GMCs did not differ between carriage-negative participants (0.78 µg/mL; 95% CI 0.63–0.97 µg/mL).

Panel B Nasal anti SPN6B IgG: When grouped by treatment arm, no significant differences in nasal IgG were observed between carriage-negative and carriage-positive participants in the placebo group at any timepoint (Fig. S1b). However, in the MV130 group Day 0 nasal IgG GMCs were higher in carriage-negative individuals (1.16 µg/mL; 95% CI 0.87–1.54 µg/mL) than in those who became carriage-positive (0.56 µg/mL; 95% CI 0.39–0.80 µg/mL). This trend continued at Day 44, with carriage-negative showing higher IgG levels (0.87 µg/mL; 95% CI 0.67–1.14 µg/mL) compared with the carriage-positive group (0.54 µg/mL; 95% CI 0.38–0.77 µg/mL. At Day 49, nasal IgG GMCs were similar between the two groups.

Panel C and Panel D Serum anti SPN6B IgG: There were no statistically different levels of anti SPN6B in serum between those participants who resisted experimental carriage (carriage negative) and those who were colonised (carriage positive), either as a group (Panel c), or when compared by treatment arm (Panel d).

Figure S5 Density of carriage determined by culture in nasal wash. Experimental carriage with SPN6B (left hand panel) and naturally acquired pneumococcal carriage density (right hand panel) by study arm and study visit.

**Figure S6** Participant compliance was assessed by weighing bottles, with a weight of 30g determined to represent full compliance. Bottle weights for participants randomised to the MV130 arm are shown in a frequency graph below. Full bottles weighed 39g and 84 sprays constituted 10g of this weight. By weight, no participants dispensed less than half of the medication.

**Supplementary Figure S7** NTR at days 0, 14, 35, 44 and 49 by study arm and sex. P-value at the top of each study visit are comparing NTR between study arms using a Wilcoxon’s rank-sum test.

**Supplementary Table 1** Neutrophilic inflammation of the nasal mucosa demonstrated by comparing neutrophil to T cell ratio (NTR) at day 0 with subsequent time-points.

| NTR by study day and arm: median (IQR) and paired Wilcoxon signed-rank p-value comparing NTR measurements from each visit to the baseline (Day 0) NTR measurements. | | | | |
| --- | --- | --- | --- | --- |
|  | **MV130** | | **Placebo** | |
| **Day** | **Median (IQR)** | **p-value** | **Median (IQR)** | **p-value** |
| **Day 0** | **0.96 (0.52,1.71)** | **ref** | **0.89 (0.53,1.51)** | **ref** |
| **Day 14** | **0.96 (0.46,1.86)** | **0.524** | **1.02 (0.73,2.69)** | **0.225** |
| **Day 35** | **1.19 (0.80,2.87)** | **0.031** | **0.96 (0.53,2.26)** | **0.581** |
| **Day 44** | **1.30 (0.63,2.83)** | **0.162** | **0.88 (0.41,2.97)** | **0.470** |
| **Day 49** | **1.59 (0.84,2.94)** | **0.026** | **1.70 (1.13,3.09)** | **0.003** |

**Supplementary Table 2** Antibody details including RRID

| **Flow Cytometry Reagents** | | | | | | | |
| --- | --- | --- | --- | --- | --- | --- | --- |
| **Marker** | **Fluorochrome** | **Clone** | **Isotype** | **Company** | **Catalogue** | **Amount per sample** | **RRID** |
| Live/Dead | APC | N/A | N/A | Invitrogen | L34973A | 5μl of a 1:500 dilution | N/A |
| EpCAM | BV605 | 9C4 | Mouse IgG2b,k | BioLegend | 324224 | 0.625μl | AB_2562518 |
| CD45 | PerCP/CY5.5 | HI30 | Mouse IgG1,k | BioLegend | 304028 | 1.25μl | AB_893338 |
| CD66b | FITC | G10F5 | Mouse IgGM,k | BioLegend | 305104 | 1.25μl | AB_314496 |
| CD3 | APC/CY7 | SK7 | Mouse IgG1,k | BioLegend | 344818 | 0.625 μl | AB_10645474 |
| CountBright™ Absolute Counting Beads | N/A | N/A | N/A | Invitrogen | C36950 | 1040/μl | N/A |

| **ELISA Reagents** | | | |
| --- | --- | --- | --- |
| **Item** | **Company** | **Catalogue Number** | **RRID** |
| Polysaccharide, purified type 6B | SSI Dagnostica | 76863 | N/A |
| Cell wall Polysaccharide Multi | SSI Dagnostica | 68866 | N/A |
| Goat anti-human IgG-AP | Southern BIOTECH | 2040-04 | AB_2795643 |
| p-Nitrophenyl phosphate substrate | SIGMA | N2770 | N/A |
| Clear Flat-Bottom Immuno Nonsterile 96-Well Plates | Thermo Fisher | 439454 | N/A |
| Tween 20 | Sigma Aldrich | P7949 | N/A |
| Human Anti-Pneumococcal capsule Reference Serum | National Institute for Biological Standards and Control, UK | 007SP | N/A |

**Supplementary Table 3** Comparison of serotype 6B carriage between study arms for compliant participants only.

|  | **All** | | **MV-130** | | **Placebo** | |
| --- | --- | --- | --- | --- | --- | --- |
| Participants n (%) | 69 | (100.0%) | 33 | (100.0%) | 36 | (100.0%) |
| **Experimental SPN6b Carriage**  Serotype 6B carriage (any timepoint) n (%) | 15 | (21.7%) | 7 | (21.2%) | 8 | (22.2%) |
| Serotype 6B carriage (day 2 post-inoculation) n (%) | 13 | (18.8%) | 5 | (15.2%) | 8 | (22.2%) |
| Serotype 6B carriage (day 7 post-inoculation) n (%) | 8 | (11.6%) | 5 | (15.2%) | 3 | (8.3%) |
