# Supplementary material for: Whole cell inactivated poly-bacterial preparation MV130 effect on nasal mucosal immunity and experimental human pneumococcal carriage: double-blind randomised controlled trial with controlled human infection model
Source: eBioMedicine. 2026 Aug 26;131:106443. doi: 10.1016/j.ebiom.2026.106443 (PMC13544312; doi:10.1016/j.ebiom.2026.106443)
Supplement: Supplementary file 3 [file mmc2.docx]

# Study Protocol

## Study title

The Influence of MV130 immunotherapy on Nasal Colonisation in a Controlled Human Infection Model of Pneumococcal Carriage in Malawi

## Short title

MV130 and Pneumococcal Colonisation in Malawi

## Reference details

**Study reference**

**NHSRC Reference**	AD23/04/4041

**Date and Version number**	2.0 09 June 2023

**Principal Investigator**	John Ndaferankhande

**Sponsor**	Liverpool School of Tropical Medicine

**Data Safety Monitoring Board**	Professor Robert C Read (Chair)

Professor John Kumwenda

Dr Mavuto Mukaka

Professor Neil French

**Trial Steering Committee**	Professor Stephen Gordon

John Ndaferankhande

Professor Jonathan Grigg

Dr Kondwani Jambo

Dr Marc Henrion

Dr Ndaziona Peter Kwanjo Banda

Dr Dingase Dula

**Funding body**	The Wellcome Trust

**Authors	Prof Stephen Gordon and John Ndaferankhande**

**Confidentiality Statement**

This document contains confidential information that must not be disclosed to anyone other than the Sponsor, the Investigator Team, and members of the Research Ethics Committee, unless authorised to do so. This information cannot be used for any purpose other than the evaluation or conduct of the clinical investigation without the prior written consent of Professor Stephen Gordon.

Contents

[Study Protocol	1](#_Toc140048860)

[Study title	1](#_Toc140048861)

[Short title	1](#_Toc140048862)

[Reference details	1](#_Toc140048863)

[Abbreviations	8](#_Toc140048864)

[Background	9](#_Toc140048865)

[Study Overview	10](#_Toc140048866)

[Main objective:	10](#_Toc140048867)

[Secondary objective	10](#_Toc140048868)

[Study Hypothesis	11](#_Toc140048869)

[Study Design	11](#_Toc140048870)

[Schedule of visits and procedures	12](#_Toc140048871)

[Study Endpoints	13](#_Toc140048872)

[Recruitment Target	13](#_Toc140048873)

[Duration	13](#_Toc140048874)

[Participants, schedule and timelines	14](#_Toc140048875)

[Method details	16](#_Toc140048876)

[Participant recruitment	16](#_Toc140048877)

[Screening and preliminary assessment	18](#_Toc140048878)

[Pneumococcal inoculation	19](#_Toc140048879)

[Randomisation	19](#_Toc140048880)

[Investigational products	20](#_Toc140048881)

[Determination of colonisation	21](#_Toc140048882)

[Safety during colonisation	21](#_Toc140048883)

[Termination of Carriage	21](#_Toc140048884)

[Immunological assays	21](#_Toc140048885)

[Analysis plan and sample size justification	22](#_Toc140048886)

[**Calculations**	22](#_Toc140048887)

[Safety considerations and assessment	23](#_Toc140048888)

[Study design to ensure safety	24](#_Toc140048889)

[MV130 safety	24](#_Toc140048890)

[Participant safety procedures	25](#_Toc140048891)

[COVID-19 specific participant safety procedures	26](#_Toc140048892)

[Evaluation of Adverse Events (AE) and Serious Adverse Events (SAE)	26](#_Toc140048893)

[Risks to researchers	27](#_Toc140048894)

[The Data Safety Monitoring Board (DSMB)	27](#_Toc140048895)

[Stopping criteria	27](#_Toc140048896)

[Ethical considerations	27](#_Toc140048897)

[Autonomy	27](#_Toc140048898)

[Non-maleficence	28](#_Toc140048899)

[Beneficence	28](#_Toc140048900)

[Remuneration	28](#_Toc140048901)

[Confidentiality and anonymity	29](#_Toc140048902)

[Samples and data	30](#_Toc140048903)

[Sample and data storage	30](#_Toc140048904)

[Collaborating laboratories	30](#_Toc140048905)

[Social Science	30](#_Toc140048906)

[Dissemination of findings	30](#_Toc140048907)

[Sponsorship and Indemnity	30](#_Toc140048908)

[Amendments	31](#_Toc140048909)

[Future development of the MARVELS programme	31](#_Toc140048910)

[Budget	32](#_Toc140048911)

[Justification of the Budget	34](#_Toc140048912)

[References	35](#_Toc140048913)

STUDY PERSONNEL

| **Chief Investigator and TSC**  **Professor Stephen Gordon**  Lead Scientist/Programme Lead | Malawi Liverpool Wellcome Research Programme,  Clinical Research Programme,  P.O Box 30096, Chichiri,  Blantyre 3, Malawi  Phone: +265 1 876444  Email: [stephen.gordon@lstmed.ac.uk](mailto:stephen.gordon@lstmed.ac.uk) |
| --- | --- |
| **Principal Investigator and TSC**  **John Ndaferankhande**  Lead clinical trial pharmacist | Malawi Liverpool Wellcome Research Programme,  Clinical Research Programme,  P.O Box 30096, Chichiri,  Blantyre 3, Malawi  Phone: +265 999329482  Email: [jndaferankhande@mlw.mw](mailto:jndaferankhande@mlw.mw) |
| **Co-Investigator (Laboratory) and TSC**  **Dr Kondwani Jambo**  Head, Viral Immunology | Malawi Liverpool Wellcome Research Programme,  Clinical Research Programme,  P.O Box 30096, Chichiri,  Blantyre 3, Malawi  Phone: +265 (0)1812423  Email: [kjambo@mlw.mw](mailto:kjambo@mlw.mw) |
| **Co-Investigator (clinical) and TSC**  **Prof Jonathan Grigg**  Paediatric Respiratory and Environmental Medicine | Blizard Institute  Queen Mary University of London  4 Newark Street  London E1 2AT  UK  Phone: 00 44 2078822206  Email: [j.grigg@qmul.ac.uk](mailto:j.grigg@qmul.ac.uk) |
| **Co-Investigator (Data and Statistics) and TSC**  **Dr Marc Henrion**  Head, Statistics Support Unit, MLW | Malawi Liverpool Wellcome Research Programme,  Clinical Research Programme,  P.O Box 30096, Chichiri,  Blantyre 3, Malawi  Phone: +265 99 333 2568  Email: [mhenrion@mlw.mw](mailto:mhenrion@mlw.mw) |
| **Co-Investigator (Clinical)**  **Dr Chimota Phiri**  Head of Department (Clinical) | Department of Medicine  Queen Elizabeth Central Hospital  P.O. Box 95  Blantyre.  Phone: +265994661629  Email: [wachimota@yahoo.com](mailto:wachimota@yahoo.com) |
| **Co-Investigator (Clinical) and TSC**  **Dr Ndaziona Peter Kwanjo Banda**  Consultant, Respiratory Medicine | Department of Medicine  Queen Elizabeth Central Hospital  Blantyre Malawi  Phone: +265 886053508  Email: [npkbanda2000@yahoo.com](mailto:npkbanda2000@yahoo.com) |
| **Co-Investigator (Clinical) and TSC**  **Dr Dingase Dula.**  Senior Clinical Doctor | Malawi Liverpool Wellcome Research Programme,  Clinical Research Programme,  P.O Box 30096, Chichiri,  Blantyre 3, Malawi  Phone: +265992892117  Email: [ddula@mlw.mw](mailto:ddula@mlw.mw) |
| **Co-Investigator (clinical)**  **Dr Anthony Chirwa**  Study Physician | Malawi Liverpool Wellcome Research Programme,  Clinical Research Programme,  P.O Box 30096, Chichiri,  Blantyre 3, Malawi  Phone: +265881858218  Email: [achirwa@mlw.mw](mailto:achirwa@mlw.mw) |
| **Co-Investigator (laboratory)**  **Dr Gift Chiwala**  Immunology and Microbiology | Malawi Liverpool Wellcome Research Programme,  Clinical Research Programme,  P.O Box 30096, Chichiri,  Blantyre 3, Malawi  Phone: +265984435022  Email: [gchiwala@mlw.mw](mailto:gchiwala@mlw.mw) |
| **Co-Investigator (laboratory)**  **Raphael Kamn’gona**  Immunologist | Malawi Liverpool Wellcome Research Programme,  Clinical Research Programme,  P.O Box 30096, Chichiri,  Blantyre 3, Malawi  Phone: +265997745692  Email: rkamn’gona@mlw.mw |
| **Co-Investigator (Clinical)**  **Edna Nsomba**  Senior Research Nurse MLW/QECH | Malawi Liverpool Wellcome Research Programme,  Clinical Research Programme,  P.O Box 30096, Chichiri,  Blantyre 3, Malawi  Phone: +265992892117  Email: [ensomba@mlw.mw](mailto:ensomba@mlw.mw) |
| **Collaborator (Laboratory)**  **Ashleigh Howard**  Research Assistant, Clinical Sciences | Liverpool School of Tropical Medicine  LLSA 01st Daulby Street, L7 8XZ, UK  Tel: +44 (0)151 705 3750  Email: [Ashleigh.Howard@lstmed.ac.uk](mailto:Ashleigh.Howard@lstmed.ac.uk) |
| **Collaborator (Clinical)**  **Prof Jonat**  Clinical Research Fellow | Liverpool School of Tropical Medicine, Respiratory Infection Group, Department of Clinical Sciences  Liverpool Life sciences Accelerator Building, 1 Daulby Street, Liverpool, L7 8XZ  Phone: 0151 705 3111  Email: [ryan.robinson@lstmed.ac.uk](mailto:ryan.robinson@lstmed.ac.uk) |
| **Collaborator (Laboratory)**  **Prof Daniela Ferreira**  Professor of Respiratory Immunology and Vaccinology | Department of Clinical Sciences,  Liverpool School of Tropical Medicine,  Pembroke Place,  Liverpool L3 5QA  Phone: +44 (0) 151 7053711  Email: [daniela.ferreira@lstmed.ac.uk](mailto:daniela.ferreira@lstmed.ac.uk) |

## Abbreviations

| AE | Adverse Event |
| --- | --- |
| CAP | Community Acquired Pneumonia |
| CFU | Colony Forming Units |
| CHIM | Controlled Human Infection Model |
| CRF | Case Report Form |
| DSMB | Data, Safety and Monitoring Board |
| EHPC | Experimental Human Pneumococcal Carriage |
| ELISA | Enzyme-Linked Immunosorbent Assay |
| ELISPOT | Enzyme-Linked Immune Absorbent Spot |
| FBC | Full Blood Count |
| GCP | Good Clinical Practice |
| HIV | Human Immunodeficiency Virus |
| HTS | Human immunodeficiency virus Testing Service |
| ICH-GCP | International Conference on Harmonisation of Good Clinical Practice |
| MARVELS | Malawi Accelerated Research in Vaccines, Experimental and Laboratory Systems |
| MHRA | Medicines and Healthcare products Regulatory Authority (UK Regulator) |
| MK | Malawian Kwacha |
| MLW | Malawi-Liverpool Wellcome Trust Clinical Research Programme Laboratories |
| MTA | Material Transfer Agreement |
| NHSRC | National Health Sciences Research Committee |
| OM | Otitis Media |
| PBMC | Peripheral Blood Mononuclear Cell |
| PCR | Polymerase Chain Reaction |
| PCV | Pneumococcal conjugate vaccines |
| PCV13 | Pneumococcal conjugate vaccine 13 |
| PCVPA | Pneumococcal Carriage in Vulnerable Populations in Africa |
| PMRA | Pharmacy and Medicines Regulatory Authority |
| QECH | Queen Elizabeth Central Hospital |
| RNA | Ribonucleic Acid |
| SAE | Serious Adverse Event |
| SOP | Standardised Operating Procedure |
| SUSAR | Serious Unexpected Serious Adverse Reaction |
| TSC | Trial Steering Committee |

## Background

*Streptococcus pneumoniae* is the leading cause of morbidity and mortality due to community acquired pneumonia (CAP), bacterial meningitis and bacteraemia worldwide (1). Pneumococcal infections cause over one million pneumonia deaths per year in children in the developing world and are also a major burden of otitis media globally. Pneumococcal conjugate vaccines (PCV) represent a great achievement in providing protection from invasive pneumococcal disease, but they are expensive to manufacture, limited in serotype coverage, associated with serotype replacement and are less effective against mucosal infection (13-20%) than against invasive disease (2).

We established a safe and reproducible Controlled Human Infection Model (CHIM) at the Liverpool School of Tropical Medicine (LSTM), U.K. This model has been used to test the effect of PCV 13versus control (Hepatitis A) vaccination in a Phase III trial (3). This model is also currently being used to test the effect of PCV 13 versus control (Normal Saline) in Malawi. This model will be used to test the effect of MV130 versus placebo. MV130 is a sublingual preparation consisting of heat inactivated bacteria strains namely Streptococcus pneumoniae 60%, Staphylococcus epidermidis 15%, Staphylococcus aureus 15%, Klebsiella pneumoniae 4%, Moraxella catarrhalis 3%, Haemophilus influenzae 3% (See Appendix 1 for Product Characteristic Sheet). Administration of the product has been seen beneficial in reducing both viral and bacterial infections through its actions in boosting long lasting humoral and cell-mediated immunity (4-7). MV130 has also been shown to induce so-called *trained immunity*, which is characterized for conferring long-lasting heterologous protection(7). In a clinical trial investigating the effect of MV130 immunotherapy on wheezing attacks in children, it was demonstrated that the MV130 showed safety and clinical efficacy against wheezing attacks (8,9). The broad nature of activity against respiratory infections, long lasting immune response and safety profile makes MV130 an appealing option in preventing pneumococcal nasal colonisation and subsequent infection.

Despite the introduction of the PCV 13 vaccine to Malawi in 2011, pneumococcal disease remains the single most important bacterial infection of adults and children (11). Long term follow-up has been rigorously carried out by the Pneumococcal Carriage in Vulnerable Populations in Africa (PCVPA) consortium (12). These data demonstrate that despite an impressive reduction in invasive pneumococcal disease for vaccinated children, there is persistent nasal carriage of both vaccine type and non-vaccine type *Streptococcus pneumoniae* in both children and HIV-infected adults (Table 1). The implications of this important finding are that (A) herd effects with PCV13 are not as strong as in Malawi compared to US data, with vaccine type pneumococcus a continued threat to vulnerable adults and children and (B) the persistent pneumococcal carriage may indicate sub-optimal or short duration of PCV13 vaccine-induced immunity. Alternative vaccine regimens are being tested (13). Cost-effectiveness for PCV13vaccination are based strongly on improved herd immunity through reduced carriage (reservoir for infection transmission) in vaccinated children (14).

| **Malawian PCVPA surveillance data** | **Children 3-6 years**  **(PCV vaccinated)** | **Children 5-10 years**  **(not PCV vaccinated)** | **HIV infected adults**  **(not PCV vaccinated)** |
| --- | --- | --- | --- |
| Vaccine type  carriage | 20.1%  (95% CI: 18.0, 22.3) | 20.1%  (95% CI: 17.5, 22.9) | 13.9%  (95% CI:11.8, 16.2) |
| Non-vaccine type  carriage | 55.9%  (95% CI: 53.3, 58.6) | 36.8%  (95% CI: 33.6, 40.0) | 29.5%  (95% CI: 26.7, 32.5) |

**Table 2:** Pneumococcal Carriage in Vulnerable Populations in Africa (PCVPA) consortium data from 2018 on pneumococcal carriage and pneumococcal conjugate vaccine 13 status (7). The data show that despite vaccination, children aged 3-6 years have the same vaccine type carriage rates as unvaccinated 5-10-year olds.

Our feasibility study of pneumococcal controlled human infection in Malawi has demonstrated no SAEs or AEs and minimal symptoms for participants who have completed the programme and experienced pneumococcal carriage for 1-3 weeks (16). Recently, our current controlled human infection model, has been used to study the effect of PCV 13 in over 200 volunteers and has shown to be safe (17). Based on successful transfer of procedures, and the conduct of the PCV 13 trial being conducted at MLW, we are confident that our clinical and laboratory procedures are sufficiently robust to test MV130 efficacy (18). Our robust social science led exploration of acceptability has demonstrated that participants feel safe, cared for and are proud to be involved with this research (19,20).

MV130 is a mixture of bacterial concentrates with 60% of the formulation consisting of different pneumococcal serotypes (1,3,5,6B,9V,14, 19F, 20 and 23F) (21,22). Testing this product in the Malawian population will allow us to compare mucosal immune responses at baseline and after MV130 stimulation and to determine if these responses are protective against pneumococcal carriage.

### Study Overview

### Main objective:

1. To examine local and systemic innate, cellular responses to MV130 compared to placebo.
2. To examine the modulation of the immune effect on MV130 by experimental pneumococcal carriage.

### Secondary objective

To determine if MV130 treatment is protective against pneumococcal carriage in healthy adults.

### Study Hypothesis

MV130 enhances mucosal neutrophil responses against *Streptococcus pneumoniae* hence inhibiting experimental pneumococcal carriage.

### Study Design

A double-blinded randomised controlled trial of adult healthy human participants experimentally exposed to 160,000 CFU/naris of *Streptococcus pneumoniae* in the nasopharynx. The investigational product will be MV130 (Bactek, Inmunotek, S.L.) and there will be a placebo. We will closely monitor study participants to ensure safety and tolerability of study procedures. We will measure immune protective responses to *Streptococcus pneumoniae* challenge using mucosal (nasal, throat and saliva) and blood samples. This study is based on the foundation of our NHSRC approved feasibility study protocol (19/08/2246) and the PCV13 and Pneumococcal carriage study protocol 16/07/2519 and will utilise standard operating procedures successfully validated in Malawi. We will indicate throughout this protocol where established feasibility **[Established, E]** study procedures are used and where there are new measures **[New, N]** from the feasibility study/PCV 13/MV130 protocol for this trial.

The study will be carried out in seven stages:

1. **Screening and recruitment:** Potential participants will be screened to ensure health and safety as detailed in the methods section **[E]**.
2. **Randomisation and treatment:** Participants will be randomly allocated to administration with MV130 or placebo**[N]**in randomised blocks (1:1 randomisation MV130 vs. Placebo) participants will receive either MV130 or placebo**.** MV130 or placebo are administered as 2 sublingual squirts (0.1ml) once daily for 42 days.
3. **Pre IMP administration samples:** Samples will be taken before MV130 treatment begins to confirm nasal pneumococcal carriage status and measure immune response **[E]**
4. **Post IMP administration samples:** Samples will be taken to measure immune response to MV130/placebo and confirm natural nasal pneumococcal carriage status **[E]**

**[E]**

5. **Inoculation:** All participants will be inoculated with *Streptococcus pneumoniae* serotype 6B to the inside of each naris. We will inoculate at a dose of 160,000 colony forming units per naris **[E]**
6. **Detection of pneumococcal carriage:** Nasal wash samples will be taken, according to a standardized protocol, at days 2, and 7 days post inoculation (23). Classical microbiological culture will determine nasal colonisation with pneumococcal serotype 6B at each time point **[E].**
7. **Immunology response measurements:** Blood, mucosal and nasal cell samples will be taken to determine the immunological response to nasal challenge **[E].**

## Schedule of visits and procedures

| **Study visit** | **A** | **B** | **B1** | **C** | **C2** | **D** | **E** | | **F** |
| --- | --- | --- | --- | --- | --- | --- | --- | --- | --- |
| **Study week** |  | 1 | 3 | 6 |  | 7 | 7 | | 8 |
|  |  |  |  |  |  |  | |  |  |
| **Day post inoculation** |  |  |  |  |  | 0 | 2 | | 7 |
| **Consent (Written)** |  | x |  |  |  |  |  | |  |
| **Consent (Verbal)** | x | x |  | x | x | x | x | | x |
| **Clinical Exam** |  | x |  |  |  |  |  | |  |
| **Vital Signs** |  | x |  | x | x | x | x | | x |
| **Medical History** |  | x |  |  |  |  |  | |  |
| **Screen for AEs** |  | x |  | x | x | x | x | | x |
| **Randomization** |  | x |  |  |  |  |  | |  |
| **MV130/Placebo^*^ administration** |  | x |  |  |  |  |  | |  |
| **Inoculation**  **HIV test** |  |  |  |  |  | x |  | |  |
|  |  | x |  | x | x |  |  | |  |
| **Pregnancy test** |  | x |  | x | x |  |  | |  |
| **FBC (2.5ml)** |  | x |  |  |  |  |  | |  |
| **PBMCs (9ml)** |  | x |  | x | x |  | x | | x |
| **Serum (2.5ml)** |  | x |  | x | x |  | x | | x |
| **Blood RNA (2.5ml)*** |  | x |  | x | x |  | x | |  |
| **Nasosorption** |  | x | x | x | x |  | x | | x |
| **Nasal Wash** |  | x | x | x | x |  | x | | x |
| **Nasal cells** |  | x | x | x | x |  | x | | x |
| **Throat swab** |  | x |  | x | x |  | x | | x |
| **Saliva** |  | x | x | x | x |  | x | | x |
| **Symptom and medication record** |  |  |  |  |  | x | x | | x |

*Table 2*

**^*Product to be administered daily for 42 days from day of visit B.^**

**Table 2: Study procedures and sampling schedule.** Study visit A may occur for up to one month before study visit B. At visit B participants will undergo the informed consent process, clinical screening and samples will be collected. These samples shall include nasal wash, nasal cells, nasosorption, urine, swabs, saliva and blood. In addition, there shall be randomization and commencement of MV130 or placebo administration if participants meet screening requirements.

Starting from visit B, the participants will have a total of 42 days treatment with MV13 or placebo and then, inoculation will occur at visit D. We will provide a window of 3 days for inoculation to occur (i.e., participants may be inoculated after taking the product for not less than 39 days or not more than 45 days). During the treatment period, there will be two clinic visits (B1 and C) prior to visit D. Visit B1 will be 14 days from visit B and visit C will be 21 days from visit B1 and 7 days before visit D. Visit C2 only occurs when participant tests positive for COVID-19 at visit B or C. More details under **COVID-19 specific participant safety procedures** (pages 23 and 24).

At Visit B1 there will be a collection of nasal wash, narsosoption, nasal cell samples and saliva. There will be a window of 3 days for each of these visits (i.e., a visit may occur at a minimum of 3 days prior or maximum of 3 days after the scheduled visit day). On these visits samples will be taken to monitor mucosal immunological response and nasal carriage. Visit E will occur 2 days after D and visit F will occur 7 days after visit D (5 days after E). At visit E there will be a health check, vital signs check (measurements of heart rate, blood pressure and breathing rate). In addition, there will also be a collection of nasal wash, nasal cells, nasosorption, swabs, saliva and blood samples. Visit F will entail the same activities as Visit E.

FBC: full blood count; PBMCs: peripheral blood mononuclear cells; RNA: ribonucleic acid. *This is for immune measures (transcriptomics) and is not a genetic test. Pregnancy test for female participants only.

### Study Endpoints

Primary endpoint **[E]:** Neutrophil count and T cell lymphocytes measurement in the nasal mucosa after 14 days of MV130 treatment.

Secondary endpoints **[E]**: Pneumococcal carriage density and duration will also be measured. Innate, humoral, and cellular responses to pneumococcal colonisation will be assessed by immunological assays on collected samples. These data will allow us to define the host variables that predict colonisation and protection due to MV130 administration.

Social science evaluation **[E]:** An exit questionnaire will be conducted as part of visit Fand to monitor participant satisfaction and acceptability of the study and procedures. This work will focus on perceptions of and concerns about controlled human infection; experience of engagement activities and consent processes; and participant recommendations for future research.

Study Setting **[E]:** Clinical procedures will be conducted at the Queen Elizabeth Central Hospital Research Ward, Blantyre, Malawi. Laboratory procedures will be conducted at the adjacent Malawi-Liverpool Wellcome Trust (MLW) Clinical Research Programme Laboratories.

### Recruitment Target

8. **[N]:** We will recruit 106 healthy adult participants that provide a full set of samples for the primary endpoint**.**

### Duration

| *MV130 STUDY GANNT CHART* | | | | | | | | | |
| --- | --- | --- | --- | --- | --- | --- | --- | --- | --- |
|  | 2023 | | | | 2024 | | | |  |
|  | Q1 | Q2 | Q3 | Q4 | Q1 | Q2 | Q3 | Q4 |  |
| Ethics and regulatory submissions |  |  |  |  |  |  |  |  |  |
| Finalise study protocols |  |  |  |  |  |  |  |  |  |
| Finalise experimental protocols |  |  |  |  |  |  |  |  |  |
| Train staff on study protocol |  |  |  |  |  |  |  |  |  |
| Participant recruitment |  |  |  |  |  |  |  |  |  |
| MV130 administration, Pneumococcal inoculation & sample collection |  |  |  |  |  |  |  |  |  |
| Data acquisition |  |  |  |  |  |  |  |  |  |
| Participant safety monitoring |  |  |  |  |  |  |  |  |  |
| Data analysis & write up |  |  |  |  |  |  |  |  |  |
| Present results locally |  |  |  |  |  |  |  |  |  |

**[N]:** Recruitment of all participants and follow up will be completed within 18 months. A Gantt chart demonstrates study activities (Figure 1)

### Participants, schedule and timelines

**[N]:** We will inoculate healthy adult participants who do not smoke tobacco or other products (including electronic cigarettes) with a well-characterised, fully sequenced penicillin-sensitive pneumococci and observe them for the development of pneumococcal carriage up to 7 days post inoculation. Study discharge will occur after visit 4 (day 7 post inoculation). The visit schedule is described in detail by Table 3.

Table 3

**Table 3:** Visit Schedule of the study

**Visit A study explanation**: (up to 1 month before screening)

**Visit B: Screening, randomization and MV130/Placebo administration**

(week 1, day 1 of MV130/placebo administration) Consent, clinical screening and samples (nasal samples, nasal cells, nasosorption, urine, swabs and blood.

Randomization and MV130 or placebo administration if participants meet screening requirements.

Visit B1: Post MV130/Placebo administration samples (day 14 of MV130/placebo administration),nasal samples, nasal cells and nasosorption.

**Visit C: Pre-inoculation samples Health check, vital signs, samples** (nasal samples, nasal cells, nasosorption, urine, swabs and blood. Vital signs include measurements, heart rate, blood pressure, breathing rate, day 35 of MV130/placebo administration)

**Visit D: Inoculation day (1 week after visit C)** Health check and vital signs. Vital signs include measurement of heart rate, blood pressure and breathing rate. Pneumococcus is experimentally put into Participant nose

**Visit E: Post inoculation samples (2 days after visit D) Health check, vital signs, samples** (nasal samples, nasal cells, nasosorption, swabs and blood. Vital signs include measurements, heart rate, blood pressure, breathing rate)

**Visit F: post inoculation samples (7 days after visit D) Health check, vital signs, samples** (nasal samples, nasal cells, nasosorption, swabs and blood. Vital signs include measurements, heart rate, blood pressure, and breathing rate)

Study visits must take place according to the proposed schedule to ensure participant safety. This will be clearly explained to the participants during recruitment and consent. If a participant is unable to comply, they will not be recruited. Daily phone contact occurs between study visits until day seven post-inoculation.

## Method details

### Participant recruitment

**[E]:** Advertisements inviting healthy participants to participate will be widely placed. Areas will include physical notice boards, table display, electronic notice boards, the intranet/internet of local universities and colleges (with permission), social media, the local press, television and radio. Staff from the MLW team will be permitted to participate. In addition, outreach events will be conducted by the study team at colleges, universities workplaces and any other places where potentially eligible participants may be found. At the outreach events, study team members will provide information about the study to a group of potential participants and address any questions or concerns.

Interested persons will be asked to contact the research team by text for further information. Once potentially interested participants have contacted the research team, they will then be invited to meet a member of the research team (information dissemination – visit A). A research team member will explain the study at the meeting and the Participant Information Sheet will be provided. Participants will then consider if they wish to continue (see Table 1).

At visit B, the informed consent process will be conducted by either a research nurse or research doctors who are formally delegated by the Chief Investigator, trained in GCP, consent and the trial protocol. Following consent, inclusion and exclusion criteria will be applied as below.

#### Inclusion criteria:

- **[E]:** Adults aged 18-40 years - ages chosen to minimise the risk of pneumococcal infection
- **[E]:** Fluent spoken and written Chichewa or English - to ensure a comprehensive understanding of the research project, their proposed involvement and communication with all members of the research team
- **[N] ]:** Access to refrigerator for the keeping of MV130/Placebo throughout the period of administration or able to come to the clinic for product administration if they have no access to refrigerator.
- **[N]**For females**,** willing to be on highly contraceptive form of contraception during the period of study participation.

#### Exclusion criteria:

- **[E]:** Previous pneumococcal vaccination/Immunotherapy or involvement in pneumococcal controlled human infection studies (inoculation with bacteria may confer immunity)
- **[E]:** HIV-infection seropositive. HIV self-test kits will be used by participants to confirm infection status in the presence of the research team. Any seropositive participant will be referred to trained personnel certified in HIV Testing Services (HTS) for confirmatory “Determine” and “Unigold” tests. Any participants confirmed as HIV-infection positive will be referred to the governmental system for infection confirmation, treatment and follow up.
- **[E]:** Close physical contact (e.g., sleeping in the same room or nursing) with at-risk individuals (children under 5 years age, immunosuppressed adults, elderly, chronic ill health)
- **[E]:** Allergy to penicillin/amoxicillin
- **[E]:** Acute illness
  - Current illness
  - Acute illness within 3 days prior to inoculation
  - Antibiotic treatment within 2 weeks of inoculation
- **[E]:** Chronic illness that may impair immune response or impair ability to comply with study procedures and safety monitoring (e.g., HIV, diabetes).
- **[E]:** Taking immunosuppressive medication that may include but is not limited to steroids and steroid nasal spray.
- **[E]:** Pregnancy - minimise risk of pneumococcal disease
- **[E]:** Involved in another clinical trial unless observational or in follow-up (non-interventional) phase
- **[E]:** History of drug or alcohol abuse. This is very difficult to ascertain in a history therefore we will exclude people reporting drinking alcohol more than twice per week.
- **[E]:** History of Smoking
  - Current regular smoker (smokes daily/ smokes > 5 cigarettes per week) - minimise risk of pneumococcal disease
  - Recent smoker i.e. within the last 6 months - minimise risk of pneumococcal disease
  - Ex-smoker with a significant smoking history (>10 pack years) – minimise risk of pneumococcal disease
- **[E]:** Unable to give informed consent
- **[E]:** In case of any uncertainty or concern, the PI will take clinical responsibility for the decision.

### Screening and preliminary assessment

Following consent, inclusion and exclusion criteria, data and samples will be collected.

- **Clinical examination** **[E]**: the initial clinic visit will include a focused clinical history and targeted clinical examination involving auscultation of the lung fields and heart sounds. Participants will be informed if their clinical examination is normal. Should a previously unrecognised abnormality be identified, this will be explained to the individual, and appropriate investigations and follow-up will then be arranged by the study team. Further participation will be determined at the discretion of the study doctor dependent on the nature of the abnormality detected. If the participant is not eligible due to an acute illness, they may be re-screened at a later date with their verbal consent.
- **Blood** **[E]**: blood samples will be obtained using venepuncture by an appropriately trained team member. A maximum of 100mL of blood will be for analysis including a full blood count. The volume of blood collected for each laboratory test is detailed in Table 1. Participants with an abnormal full blood count will be excluded from the study as determined by the clinical team.
- **Nasosorption strips [E]:** Nasosorption will be obtained before the nasal wash. The strips are similar to blotting paper and developed by Hunts Development Ltd (UK). Strips collect concentrated nasal lining fluid before the nasal wash to measure inflammatory responses that may be associated with increased colonisation density and acquisition. Concentrated nasal fluid will be used to measure cytokine levels by multiplex bead array. Blotting paper will be held inside the nostril for up to 3 minutes until soaked. These will then be removed and placed in a microcentrifuge tube for storage.
- **Nasal wash** **[E]:** will be performed using a modified Naclerio method (23). This is a well-used and validated technique to collect nasal bacterial specimens used successfully during our feasibility study. Briefly, 5ml of sterile saline is instilled and held for a few seconds in the nares before being expelled into a sterile Gallipot. This is repeated twice in each naris using 20ml saline in total. In the event of nasal wash loss (defined as cough/sneeze/swallow), the procedure may then be repeated to obtain an adequate specimen.
- **Nasal** **Cells [E]:** will be collected using a nanosampling method in which cells are obtained through minimally invasive superficial nasal scrape biopsies (rhinoprobe) (18). Nasal cell samples will be obtained after nasal wash samples have been taken. Up to 4 samples (2 per nostril) will be obtained at each nasal sampling visit. If the researcher finds that the sample is insufficient, for example no cells are visible on the rhinoprobe, the sample can be repeated immediately. The rhinoprobes are placed in transport media immediately after collection.
- **Throat swabs [E]:** simple posterior pharyngeal swabbing on a dry swab and placed into culture media. This test will be used to determine viral co-infection (including COVID-19).
- **Saliva [E]:** salivette held for saturation or a spit in the tube.
- **Urine Sample [E]:** for a pregnancy test as part of the safety screening.

### Pneumococcal inoculation

#### Preparation of bacterial stock and inoculation

- **Initial preparation and transfer to MLW [E]:** Microbiological and sequence confirmation of the purity of the inoculum will take place at LSTM and reference Public Health England laboratories prior to the onset of the study. Once complete, the microbiologically and genetically confirmed *Streptococcus pneumoniae (S. pneumoniae)* capsular serotype 6B strain BHN418 will be transferred from the LSTM to MLW. Microbiological and serotype confirmation will take place at MLW upon receipt.
- **Preparation of bacteria for carriage studies at MLW** **[E]:** LSTM will provide MLW with mid-log broth cultures of pneumococcus, frozen at -80°C in aliquots of glycerol-enriched media. Immediately prior to participant inoculation, aliquots will be thawed, washed twice, and re-suspended in sterile saline at an appropriate density for each inoculation dose. Our validated [E] Standard Operating Procedure (SOP), based on LSTM protocols, will be used.
- **Confirmation of accuracy [E]:** Following culture by the laboratory team at MLW, aliquots of the bacterium will be returned to the UK, as a quality control measure, to confirm the identity, purity and penicillin sensitivity of the isolate. These bacterial isolates will be sequenced in the UK to confirm identification.
- **Regulatory approval [E]**: *S. pneumoniae* transfer will be reported to the Malawian Pharmacy and Medicines Regulatory Authority (PMRA, national regulatory authority) prior to initiation of the study. MV130 and Placebo are investigational medicinal products and will require PMRA review and approval during the application process [N].
- **Inoculation** **[E]**: using a P200 micropipette, 0.1ml saline containing the desired dose of pneumococcus will be instilled into the nose. The participant will be seated in a semi-recumbent position. After inoculation, the participant will remain in this position for up to 15 mins. They will be given a post-inoculation advice sheet (including emergency contact details), thermometer, a course of amoxicillin and a daily symptom log to complete.
- **Dose administration [E]:** We will administer the inoculation dose of 160,000 CFU/naris. The 160,000 has been safely used in Liverpool and Malawi with no adverse events (24).

Randomisation

**[N]:** Participants will be individually randomized to either MV130 (Bactek) or Placebo prior to primary inoculation. A random number generator will be used to generate the randomization sequence and randomization occurs using block randomization with random block sizes of 6, 8 and 10 to ensure balanced vaccine allocations. An independent statistician will run the randomisation code for study group allocations and allocations will be stored on a secure electronic portal at MLW. The research team will be blinded to the treatment group.

Procedure: The pharmacy team will be fully unblinded and prepare each will receive the numbered treatment according to the randomisation code. Individual IMP kits will be prepared to maintain research team and participant blinding. Individual IMP kits will be identified only by a randomisation identification number and barcode. The randomisation number assigned to each subject will coincide with the number of the investigational product they will receive. This number will always be the lowest number still available at the site and printed on the treatment box as the treatment number. Unblinding by the investigators will occur once microbiological reporting is completed for the final participant at visit F (7 days after primary inoculation).

### Investigational products

**[N]:** Both the MV130 and Placebo will be supplied to MLW by immunotek, S.L.Spain. MV130 formulation contains as active ingredient/s different species of whole inactivated bacteria at 300/FTU/ml (equivalent to 10^9^ bacteria/ML) which are frequently present in the respiratory tract: V102 *Staphylococcus aureus* (15%), V101 *Staphylococcus epidermidis* (15%), V104 *Streptococcus pneumoniae* (60%), V113 *Klebsiella pneumoniae* (4%), V105 *Moraxella catarrhalis* (3%) and V103 *Haemophilus influenzae* (3%). Excipients include glycerol, pineapple artificial flavouring, sodium chloride and water for injection. Placebo contains only the excipients. The dosage is 2 sprays dily (in a single procedure, one dose is 2 sprays) for a final daily dose of 0.2ml. The products will be stored at the appropriate temperature and administered as per manufacturer’s instructions. Both the MV130 and Placebo will be stored under refrigeration between 2℃-8℃. They will be administered taking two sprays under the tongue per day in a single procedure, resulting in a final daily dose of 0.2ml. The first dose of the products will be administered at the clinic under the supervision of expert study staff but the consequent doses will be administered by the participants themselves. The administration duration will be a total of 42 days. The administration of MV130/Placebo will be as follows;

1. Remove the plastic seal from one bottle.
2. Gently shake the bottle.
3. Rotate the nozzle sideways. Spray 3 or 4 times to ensure the pump mechanism fills up (only

when starting the bottle).

4. Lift your tongue and direct the nozzle under your tongue and spray twice to apply the

appropriate dose.

5. Retain the dose under your tongue, for approximately 1-2 minutes before swallowing.
6. Rotate the nozzle back to its original vertical position, thereby locking the spray mechanism.
7. Return the bottle back to the box and store in the fridge (2^0^C-8^0^C). Avoid sudden temperature changes.

The IMP must be administered as separate as possible from the intake of food and drinks (at least, 30 minutes prior and after the consumption of food or drink). Brushing the teeth or rinsing the mouth should be avoided 30 minutes prior or after the administration of the IMP.

The Technical Director of Immunotek S.L. guarantees the manufacture, the labelling and adequate quality of the IMP, according to Annex 13 of Good Manufacturing Practice (GMP).

The IMP will be supplied in a resistant plastic box, containing 2 amber glass bottles, closed with a white plastic applicator with an integrated pump spray inserted. The participants will be asked to return the unused bottles at visit B as one way of checking compliance.

### Determination of colonisation

**[E]:** Colonisation will be defined by the microbiology result of nasal washes taken at 2-, and 7- days post inoculation. Nasal washes will be plated on to culture media and incubated overnight at 37°C in 5% carbon dioxide (CO_2_). Colonies will be confirmed as *S. pneumoniae* using classical microbiological techniques including (i) typical draughtsman-like colony morphology, (ii) the presence of α-haemolysis, (iii) optochin sensitivity and (iv) bile solubility. Typing by latex agglutination will be done using a commercial kit to confirm pneumococcal serogroup. Isolates will be frozen at -80^o^C for storage. Results from the cultured nasal wash will also be confirmed using Polymerase Chain Reaction (PCR) based methods of bacterial detection.

**[E]:** Monitoring of colonisation will be performed by microbiology analysis of nasal washes. This will include measurement of pneumococcal density by LytA PCR. Monitoring for COVID-19 co-infection will be performed by RT-qPCR analysis of throat swab samples

### Safety during colonisation

**[E]:** A three-day course of amoxicillin and a digital thermometer will be given on the day of inoculation. These will be carried by the participants for immediate treatment after phone consultation should the participant develop moderate or severe symptoms as described on the participant safety advice leaflet. Participants will be required to make text message/phone contact with a specified member of the research team before 1200 hrs every day for 7 days post-inoculation *regardless of whether they have symptoms or not*. Should they not make contact by the specified time, a member of the research team will contact the participant. If no contact is made, then a ‘secondary contact’ (established during screening visit) will be telephoned. During the post-inoculation period, participants will have access to a 24/7 on-call telephone service until the end of the study.

### Termination of Carriage

**[E]:** All study participants who are nasally colonised with pneumococcus serotype 6B at the end of the study will be asked to take oral amoxicillin 500mg three times daily for 3 days. This will be communicated at visit F, or by telephone in the unlikely event of only visit samples (day 7) showing carriage.

### Immunological assays

- **Antibody titres [E]**: measurement of pneumococcal-specific protein and polysaccharide antibodies will be conducted on nasal wash, salivary and serum samples using ELISA. In addition, we will also measure lysozymes, lactoferrins and beta-defensins. These will allow us to identify potential antibody correlates of protection against pneumococcal colonisation.
- **Cellular responses [E]:** measurement of pneumococcal-specific memory B and T cells will be performed on peripheral blood and nasal samples using flow cytometry and ELISPOT. In addition, neutrophils will also be measured on the peripheral and nasal mucosa samples. This will allow us to identify potential cellular correlates of protection against pneumococcal colonisation.
- **Host transcriptomic analysis [E]:** measurement of host nasal mucosa transcriptomic profile will be done using RNA sequencing (RNAseq) on nasal cells and peripheral blood. This will allow us to characterise potential nasal or blood transcriptomic signatures associated with protection or susceptibility to pneumococcal carriage.
- **SARS Cov 2 spike protein testing (serum) at visits B, C, E, and F. 100ul of serum will be required for this testing.**

### Analysis plan and sample size justification

The primary endpoint will be the neutrophil to T-cell ratio (NTR). This is because the NTR is a good measure of mucosal inflammation (19).

The primary efficacy analysis will compare log (NTR) between MV130 randomised individuals and controls at the inoculation visit using a standard two-sample t-test. A secondary analysis will be adjusted for individual participant characteristics that could impact the endpoint and which may be imbalanced between treatment arms**.** To inform our sample size calculations, we investigated the distribution of NTR and log (NTR) measurements observed in the MARVELS PCV-13 trial, in the CFU160, 000 dose group(24, 25).

From the MARVELS PCV-13 trial, there are no major distributional differences between study visits and vaccination groups. The overall standard deviation for log(NTR) is estimated as σ =1.2.

The following assumptions have been made with regards to the calculation:

- We assume a standard statistical significance level of 5%:

We aim for a minimum power of 80%:

- We assume independent data observations.
- We assume that the log(NTR) measurements are not characterised by severe non-normality so that we can assume the sample means to be approximately normally distributed (guaranteed by the Central Limit Theorem unless severe non-normality, such as extreme outliers, is present in the data).
- We assume a standard deviation of σ =1.21 for log(NTR) common to both treatment groups. This is the estimate we observed in the MARVELS PCV-13 trial data.

Total sample size is limited to, i.e., 50 in each group assuming a 1:1 randomisation ratio.

We assume that 6% of sample will not be able to be processed (for a variety of participant and lab technical reasons), meaning that we would recruit 106 participants to have useable data on 100 participants.

**Calculations**

Two-sample t test power calculation

n = 50

delta = 0.6862194

sd = 1.212651

sig.level = 0.05

power = 0.8

alternative = two. sided

NOTE: n is number in *each* group

With the above assumptions, the study would be able to detect a difference in log(NTR) between both groups of 0.69 with 80% power. This corresponds to a standardised effect size of 0.57 which is typically regarded as a moderate effect size.

With the caveat that the average of a log is not the log of an average (meaning the calculation that follows is not correct and should only be viewed as a very rough guide), this corresponds to a relative change in NTR of 98.62% (i.e., slightly more than a doubling).

Participant consent is required before screening procedures can be conducted. Any participant who consents but is subsequently determined to be ineligible will be replaced so that a total of 106 participants will be inoculated with *Streptococcus pneunoniae* for the study*.* Important secondary endpoints including the immune responses, in particular humoral and cellular responses will also be assessed. Immunological parameters will be compared between pre-vaccination and pre-inoculation values in paired analyses using parametric or non-parametric tests as appropriate.

## Safety considerations and assessment

Reporting of AEs and SAEs

To assess safety, all AEs and SAEs occurring during the course of the trial, from the time of signature of the informed consent, to the completion of the trial will be recorded and evaluated. The Division of AIDS (DAIDS) Table for Grading the Severity of Adult and Pediatric Adverse Events version 2.1 July 2017 (Supplementary materials 1) will be used to assess severity and relatedness of all symptoms, signs and abnormal laboratory results to inoculum or study product. Severity (intensity) of AEs will be graded from grades 1-5 as follows: Grade 1: mild event, Grade 2: moderate event, Grade 3: severe event, Grade 4: potentially life-threatening event and Grade 5: death. Relatedness to inoculum or study product will be graded as: Not related, Possibly related, Probably related or Definitely related. AEs will be assessed by a research clinician and reported to the study database as soon as they occur. The MARVELS AE form version 1.0 dated 17 Feb 22 (Supplementary materials 2) will be used to report and track all AEs.

Death, a life-threatening event, an event resulting in or prolonging hospitalisation, disability or permanent damage, or any event that requires intervention to prevent permanent impairment or Damage or any other serious or medically important event will be regarded as a Serous Adverse Event (SAE). Upon site awareness of an SAE, the DSMB and ethics will be informed within 24 hours. The study will be temporarily held pending direction from the DSMB. The study SAE form (Supplementary materials 3) will be used to record SAEs into the study database. Relatedness of an SAE to study product or inoculum will be assessed as described above.

Suspected unexpected serious adverse reactions (SUSARs) that occur in this clinical trial, that are not listed in the MV130 product information sheet, and meet one of the serious criteria, such as death, life-threatening, hospitalization, disability, or birth defect will be reported within 24 hours to the DSMB, REC's and study sponsor. Recruitment in the study will be on hold, pending DSMB advice, in order to protect other trial participants from unknown or undocumented risks. SUSAR reporting will be done electronically via email, and hard copies of all reports filed in the participant's binders and study Investigator's Site File.

### Study design to ensure safety

**[E]:** Safety is paramount in human infection studies. While the risk to individuals of developing any infection is very low (40% Malawian adults experience natural colonisation at any time, and the incidence of invasive disease is 20/100 000 patient years (26)), the study is designed to ensure any risk is minimal by appropriate:

- Study team selection and rigorous training in human challenge procedures
- Study design with staggered recruitment approach (sample size)
- Careful serotype selection, demonstration of safety and acceptability in our feasibility study and the MARVELS PCV 13 study and 10 years of pneumococcal controlled human infection experience at LSTM
- Participant selection and exclusion criteria as detailed
- Participant education through a participant information sheet (provided)
- Rigorous safety procedures including daily monitoring and providing participants with a thermometer and a course of amoxicillin tablets in case of emergency
- 24-hour emergency telephone contact with researchers, including close individual daily monitoring, and access to hospital facilities and prompt treatment if required
- To further mitigate risk, participants will be provided with accommodation for three nights immediately following nasal challenge. If a participant determines that it is not possible to stay at study accommodation for three nights, then a pragmatic approach to further study procedures will be taken. If the participant can demonstrate that they remain readily available by phone and accessible with transport, this need not affect the procedure. Twice daily checks could replace the resident period. In the Liverpool CHIM there is no resident period at all. If, however, the participant absconds or is intending to be uncontactable, then participation in the study will be stopped and antibiotics offered as at the end of the protocol.
- In the event that a study participant became unwell due to pneumococcal infection during the course of the study, in addition to treating the participant, we will offer an appointment to immediate household members to perform a medical check-up and provide reassurance.

The research investigators have successfully worked together to safely deliver our pneumococcal CHIM feasibility study in Malawi based on knowledge and experience from the Liverpool studies (27). In addition, there is also knowledge based on the MARVELS PCV 13 trial (15). No episodes of pneumococcal infection or serious unexpected serious adverse reactions (SUSARS) have occurred in any of our participants whether they had no carriage, natural carriage or experimental carriage.

### MV130 safety

**[N]:** MV 130 has proven to be safe previously in a number of studies. In one study in which it was used for six months on a daily basis, with a subsequent six month follow up, there was no adverse effects related to treatment (11). Likewise in another study in which the MV130 was used together with antibiotics to investigate its effect on tonsillitis, there was also no adverse reactions linked to MV130 observed (21). In addition, in three pilot retrospective studies carried out in vulnerable population including patients with common variable immunodeficiency, B cell haematological malignancies or subjects with autoimmune diseases no adverse reactions were reported(7-8,22).

In general, the adverse reactions with MV130 are very rare. Local reactions (oropharyngeal) have been reported and some other systemic one; asthenia, rash and pruritis but very rarely (Summary of product characteristics, appendix). Cases of asthma and bronchospasm have also been reported before but very rare (<1/10000) (Summary of product characteristics, appendix).

### Participant safety procedures

**[E]:** We will provide all participants with a safety information sheet (enclosed within this submission pack). This sheet classifies symptoms into mild, moderate and severe. We will take a proactive approach for participants, with daily contact up until day seven post inoculation and enquiry about development of any symptoms. We will also encourage participants to report development of any symptoms with 24 hour medical cover throughout the study visit schedule. In the event of illness, we will apply standardised operating safety procedures for participants. The research team will pay for any costs associated with these procedures and participants will incur no out of pocket expenses.

#### Mild illness

**[E]:** Mild illness is defined as development of any new symptom that is of concern to the participant during the study period. These may include mild coryzal symptoms such as blocked or runny nose. Participants will be encouraged to contact the research team by telephone within office hours. During the telephone assessment participants will be screened for potentially concerning moderate and severe symptoms. If there is any concern, participants will be invited to the research clinic for a detailed clinical assessment by one of the study doctors. The assessment will be fully documented within study records including rationale for any treatments and/or required modifications to study procedures.

#### Moderate Illness

**[E]:** Moderate illness events are defined as fever with temperature >37.5˚C, shivering, headache, new rash, drowsiness, cough, new earache and or new eye infection. Participants will be clearly instructed to call the research team at any time of the day upon development of any of these symptoms. During the telephone assessment participants will be screened for severe symptoms and requested to attend either the research clinic (office hours) or Mwaiwathu hospital (outside of office hours) for medical assessment. If severe symptoms are identified on telephone assessment, participants will be asked to take antibiotics from their supplied emergency pack and attend Mwaiwathu hospital. Study doctors will assess the participants at either the research clinic or Mwaiwathu and offer appropriate treatments. The assessment will be fully documented within study records including rationale for any treatments.

#### Severe illness

**[E]:** Severe illness events are defined as any symptoms that cause serious concern to participants following inoculation. We have purposefully taken a conservative approach to encourage participants to seek early medical advice and treatment if they are concerned. Participants will be counselled as to what symptoms the study team may be particularly concerned about during the consent process. These would include any potential symptoms of sepsis. In the event of severe illness, participants are requested to start taking the provided emergency antibiotics, directly attend Mwaiwathu hospital and contact the study team. A study doctor will attend the patient in Mwaiwathu and offer appropriate treatments. The assessment will be fully documented within study records including rationale for any treatments.

### COVID-19 specific participant safety procedures

***Participants who test positive for SARS-CoV-2 at visit B (screening and vaccination visit)***

If a participant tests positive for SARS Cov-2 at screening (visit B), they will remain in the study and advised to isolate for a minimum of 10 days. They will be scheduled an inoculation preparation visit (visit C) within 11 weeks of the day of occurrence of visit B per protocol. Participants with clear SARS-CoV-2 infection within these 11 weeks and can be safely reviewed in clinic for visit C. Repeat SARS-CoV-2 testing will be performed at visit C together with other routine visit C procedures.

***Participants who test positive for SARS-CoV-2 at visit C (inoculation preparation visit)***

There will be an allowance of a single repetition of visit C if a participant was ineligible for inoculation during the first visit C due to positive SARS-CoV-2 result. After an isolation period of 10 days (flexibility up to 14 days) the participant will be called back to the clinic where repeat visit C (C2) including SARS-CoV-2 testing will be completed. If repeat SARS-CoV-2 test is negative, the participant will be scheduled for an inoculation visit (D) to be performed within 7 days of visit C2. If the repeat SARS-CoV-2 test remains positive, the participant will be withdrawn from the study and followed up per standard of care. The repeat visit C will be performed within 11 weeks of enrolment. If a repeat visit C cannot be performed within 11 weeks of enrolment, the participant will be withdrawn from the study.

***Participants who test positive for SARS-CoV-2 at visits E, and F (post-inoculation visits)***

If a participant tests positive for SARS-CoV-2 at visit E (day 2 after inoculation), they will remain in the study and isolate for 10 days. They will be called on day 14 post inoculation for a visit E (study exit) where all visit E procedures, including SARS-CoV-2 testing will be performed.

Participants that test positive for SARS-CoV-2 at visit F (study exit) will be advised to isolate at home for a minimum of 10 days.

If the participant has concurrent pneumococcal carriage, they will be advised to commence antibiotics to reduce the theoretical risk of bacterial co-infection.

We will replace participants who are excluded during post-inoculation follow up to recruit the pre-specified allocated number of patients to each group.

### Evaluation of Adverse Events (AE) and Serious Adverse Events (SAE)

#### Adverse Events

**[E]:** Non-serious adverse events will be collected systematically during the research and recorded in the case report form. The procedure for carrying this out will be the same as in “Safety considerations and assessment” section. Participants will keep a log of symptoms and this will be summarised and reported to the DSMB.

#### Serious Adverse Events

**[E]:** Any SAE as defined in ICH-GCP occurring to a research participant will be reported to the DSMB, NHSRC and study sponsor within 24 hours of the study team becoming aware of the event. In this event, the research will be stopped temporarily for investigation and any further work deferred until DSMB and NHSRC advice has been provided to the TSC for consideration. A follow-up report will be submitted within 24 hours of any change/resolution of the condition.

### Risks to researchers

**[E]:** These are standard clinical (needle stick) and laboratory (biohazard) risks. Experienced staff will carry out procedures that are within their competencies in accordance with standard operating procedures regulated by good clinical practice and national guidelines. Appropriate risk assessments are in place for all laboratory SOPs. All laboratory work will be conducted in an appropriately rated laboratory in line with health and safety regulations for research with human tissues / infectious agents. All staff will be provided with and training in the appropriate use of personal protective equipment to reduce the risk of COVID-19 infection. We will use UK Public Health England PPE guidance recommendations (21).

### The Data Safety Monitoring Board (DSMB)

**[E]:** The DSMB will be established prior to commencing this study and include members expert in controlled human infection, safety and statistical procedures. Prof Rob Read will Chair - Prof Read has chaired the LSTM DSMB for 10 years.

The DSMB will monitor the study and advise the TSC, including the PI and study team. Briefly, all SAEs will be reported to the DSMB and study sponsor within 24 hours and recruitment/inoculation paused pending DSMB review and recommendation to the Trial Steering Committee (TSC) in line with GCP guidelines. The study team will provide at least monthly update (by email) on all recruitment to the DSMB. The DSMB will meet formally (by telephone conference) biannually and in the event of any SUSARs.

## Stopping criteria

**[E]:** This study will pause in the event of a Serious Adverse Event or on completion of the Protocol. Thereafter, DSMB, PMRA and NHSRC members will assess the event before advising the Trial Steering Committee on measures required to resume the study or if the study should be stopped. The study will only be resumed if the DSMB, PMRA and NHSRC are satisfied that required measures are in place. If the DSMB, PMRA or NHSRC recommends the early stopping of the study, all reasons must be stated clearly. If there are DSMB, PMRA or NHSRC members with dissenting views, these must be appended to the recommendations. If there are no trial related serious adverse events, the study will stop upon completion of the protocol.

## Ethical considerations

### Autonomy

**[E]:** The participants will be given high-quality information that is written and spoken using common lay terms without jargon to allow them to understand the research objectives and the risks and benefits of all procedures. They will then be given time to consider the information before consenting to any involvement. At no stage should the participant feel pressured or persuaded into participating in the research study.

Participants have the right to withdraw their consent and therefore withdraw from the study at any time without giving reason. If a participant withdraws from the study, we will recruit an alternative participant to complete the study.

### Non-maleficence

**[E]:** Inoculation of *S. pneumoniae* will be as per the established and safe protocols and will be performed by highly trained staff with close supervision (24hr on call access to medical professionals involved in the study) and follow-up. Specific inclusion and exclusion criteria are set to further protect the participant. Experience and trained research staff will perform venepuncture and nasal sampling.

### Beneficence

**[N]:** Participants will receive a health check including HIV-infection status assessment when taking part in this study. Participants also have the potential to benefit from completed MV130 administration schedules during the study. In addition, participants may benefit from a better understanding of clinical research, they may also benefit from a sense of contributing to medical research in a valuable way. Participants will be remunerated for their time and inconvenience – discussed below.

## Remuneration

**[N]:** It is intended that financial factors will not significantly influence an individual's decision to participate in this study. We reimburse participants for out of pocket expenses such as travel, communication and compensate time spent and burden. The sums offered in this study are consistent with remuneration guidelines published in Malawi, paid pro-rata (per activity and not dependent on study completion) (22).

If a participant withdraws from the study early, they will be compensated for the parts they took part in up until the time they withdrew. Payments are summarised in Tables 4 and 5, MK 16,500.00 will be paid at the end of study visits B, C, C2 (*if applicable*), D, E, and F.

| Visit(s) | Procedure (Reimburse expenses) | Duration(min) | Payment/visit (MK) |
| --- | --- | --- | --- |
|  |  |  |  |
| A | Study information, eligibility questions, informed consent, | 60 | 0 |
| B | Medical history, clinical examination, vital signs, samples (nasal wash, nasal cells, urine, throat swab, blood), IP receipt | 60 | 20,000 |
| B1 | Post MV130/Placebo administration samples (nasal wash, nasal cells, nasosorption) | 45 | 20,000 |
| C | Medical history, clinical examination, vital signs, samples (nasal wash, nasal cells, urine, throat swab, blood) | 45 | 20,000 |
| C | Accommodation (one night) | 1 day | 35,000 |
| C | Subsistence (one meal per night) | 1 day | 35,000 |
| D, | Health check, Samples (nasal wash, nasal cells, throat swab) | 20 | 20,000 |
| E | Health check, Samples (nasal wash, nasal cells, throat swab) | 20 | 20,000 |
| F | Health check, Samples (nasal wash, nasal cells, throat swab) | 20 | 20,000 |
|  | TOTAL |  | 190000 |

**Table 4: Research participant remuneration. Accommodation/meals and board costs will be paid directly by the research team. Participants will not receive compensation to attend the information visit (Consent). Visits A-F will cause mild/moderate discomfort. In addition, participants who will have their product administration at MLW on a daily basis will receive a transport refund of K5000 for each trip.**

**Participants will be provided remuneration by direct electronic bank transfer (if possible, else cash) at each visit.**

## Confidentiality and anonymity

**[E]:** Only authorised members of the research team will have access to any personal information. Only information of direct relevance to the study will be collected. All electronic records containing personal information will be stored in a password protected database on a password protected server at MLW. Electronic case report form data will be collected on encrypted study-specific tablet devices and synchronised daily onto the MLW server. This approach is well established at MLW, and the policy governing MLW data management policy is available upon request. Paper documentation containing personal information will be kept in a locked filing cabinet in a locked room in the Queen Elizabeth Central Hospital research clinic.

Each participant will be assigned a unique non-identifiable study number by a member of the clinical research team at recruitment. Unlinked non-identifiable clinical data will be stored and analysed at the MLW-laboratories and collaborating laboratories.

For the exit interview the following ethical considerations will be applied to ensure privacy and confidentiality is upheld. Following each interview, audio files will be transcribed verbatim into written Chichewa or English (depending on language used in interview) and translated to English where necessary. The research assistant responsible will save the audio file on a secure cloud-based network drive that will only be accessible to the social science team of researchers. Any paper copies will be stored in a locked filing cabinet.

Participant names will not be included in transcripts or file names; instead participants will be given ID numbers. Enrolment logs and any consent documents that include participant names or contact details will be stored separately in a locked filing cabinet. Electronic recordings and transcripts will be stored on a secure cloud-based drive for a minimum of 5 years from the end of data collection, to allow data analysis, publication, and ability to the wider scientific community to check our results.

## Samples and data

### Sample and data storage

**[E]:** MLW will act as custodian for all data and samples collected during the study. Consent will be obtained from the participant to use the samples for this research only. Samples will be stored for a maximum of five years.

### Collaborating laboratories

**[E]:** Samples will also be sent to national and international collaborating laboratories (MTA attached with application) to utilise specialist expertise not available in Malawi. Samples will be labelled with the anonymised study number. Consent will specifically be obtained from the participants to allow samples to be sent to our collaborators.

## Social Science

**[N]:** All participants will be asked to complete a digital questionnaire upon completion of study related activities at the end of visit F. If a participant expresses any concerns or dissatisfaction, they will be offered a follow up visit to explore the issue(s) in more detailed with a trained qualitative researcher. The interview will take place on a separate date and location and with a qualitative researcher who has not been involved in clinical delivery of the study. The interview will be semi-structured and focused on concerns raised by the participant, lasting approximately 30minutes. Participants will be offered additional compensation for time and travel to this interview.

## Dissemination of findings

**[E]:** The findings from this study will be disseminated amongst the scientific community. We intend to publish our findings in peer reviewed scientific journals and present data at appropriate local, national and international conferences. We will produce a close-out report for the NHSRC, PMRA and LSTM (sponsors) at the end of the study and a final report once data are published. In addition, we will produce a lay report of our findings which will be made available to all participants.

## Sponsorship and Indemnity

**Sponsorship [E]:** The Liverpool School of Tropical Medicine (LSTM) has provided an intention to sponsor letter provided with this application.

**Indemnity [E]:** We will purchase no fault clinical trials insurance to cover research participants for this study through LSTM.

## Amendments

**[E]:** Future amendments to the protocol will require approval by the National Health Research Ethics Committee, PMRA, and the Sponsors.

## Future development of the MARVELS programme

**[N]:** Success in this project will result in:

A) An understanding of the immunological mechanism induced by the administration of MV130.
B) In-country capacity building to conduct future Immunotherapy testing studies. Immunotherapeutic agents are becoming the promise of target specific therapies with reduced side effects.

Future work will be planned to build on both of these anticipated outcomes by engaging with vaccine manufacturing companies and mucosal adjuvant programmes to develop more efficacious pneumococcal vaccines and immunotherapeutic agents for the Malawian population.

## Budget

| **Summaries** | **Total (£)** | **Total (MK)** |
| --- | --- | --- |
| Salaries | 0 | 0 |
| Research materials and consumables | 90494 | 117642200 |
| Equipment | 9000 | 11700000 |
| Miscellaneous | **66473.08** | **86415000** |
| **Total** | **165967.08** | **215757200** |
| **Research materials and consumables** | | |
| Nasal, throat, Saliva Immunology | 23800 | 30940000 |
| COVID-19 testing | 7840 | 10192000 |
| Clinical test (Pregnancy, FBC, HIV) | 24000 | 31200000 |
| Nasal microbiology | 20354 | 26460200 |
| Stationery, Printing, Photocopying | 1000 | 1300000 |
| Servicing lab and clinic equipment | 2000 | 2600000 |
| Participant transport and expenses (Malawi) | 2500 | 3250000 |
| Shipping (WTTC) and clearing costs (MLW) | 5000 | 6500000 |
| MLW transportation costs (Field Research Assistant, Airport transfers | 4000 | 5200000 |
| **Total Research materials and consumables** | **90494** | **117642200** |
| **Equipment** | | |
| IT equipment licences, software | 500 | 650000 |
| Computer accessories (gadgets, servicing) | 500 | 650000 |
| Cool boxes | 6000 | 7800000 |
| Data loggers | 2000 | 2600000 |
| **Total Equipment** | **9000** | **11700000** |
| **Miscellaneous** | | |
| Staff training | 750 | 975000 |
| Staff uniforms & registration | 250 | 325000 |
| Telephone communications | 2000 | 2600000 |
| Transport costs | 600 | 780000 |
| Sciences and public communication costs | 3000 | 3900000 |
| Data entry and statistics costs | 12000 | 15600000 |
| Health and safety including PEP | 500 | 650000 |
| NHSRC processing costs | 150 | 195000 |
| DSMB/LSM/TSC | 4000 | 5200000 |
| Study participants compensation | 17123.08 | 22260000 |
| Study participants accommodation costs | 8000 | 10400000 |
| No fault insurance for study participants and fronting | 10000 | 13000000 |
| Archiving costs | 100 | 130000 |
| Travel and subsistence | 2000 | 2600000 |
| Conference fees | 2000 | 2600000 |
| Other travel and subsistence | 4000 | 5200000 |
| **Total Miscellaneous** | **66473.08** | **86415000** |
| Additional regulatory costs | | |
| **NHSRC Human Subjects Protection** | **16, 596.71** | **21,575, 720** |
| **PMRA application, review and registration of clinical trial** | **8, 298.35** | **10, 787,860** |

## Justification of the Budget

**Salaries:** Skilled staff in adequate numbers are critical for success in human infection studies. We will use existing MARVELS staff to conduct the trial. Salaries were already secured in the current MARVELS grant up to December 2024.

**Research materials and consumables:** Participant samples will be processed in laboratories at MLW. Consumable costs for Immunology (blood, nasal, throat and saliva - £23, 800), Microbiology (nasal- £20,354,)) and Clinical (FBC, HIV and pregnancy - £24, 000) samples are detailed in the budget. In addition, we have detailed standard infrastructure, inoculum shipping and transport costs in this section. We will also pay for COVID-19 tests for participants, the unit cost per test is £10 with six tests required.

**Equipment:** Minimal additional equipment is required as this has previously been purchased for our feasibility study and the PCV13 trial.

**Miscellaneous:** The major miscellaneous items relate to development of our electronic data entry system and statistics costs (£12,000), costs to support DSMB and TSC support for the project (£4,000) and insurance fronting for the research project (£10,000). In addition, we have included participant compensation (£17,123.08) and subsistence (£2000) and communication costs (public engagement, telephone - £3000),

As a Wellcome funded project, we partner the KUHeS in many ways including training (£750). We have allocated £16, 596.71 to NHSRC human subject protection).

## References

1. Levine OS, O'Brien KL, Knoll M, Adegbola RA, Black S, Cherian T, Dagan R, Goldblatt D, Grange A, Greenwood B, Hennessy T, Klugman KP, Madhi SA, Mulholland K, Nohynek H, Santosham M, Saha SK, Scott JA, Sow S, Whitney CG, Cutts F. Pneumococcal vaccination in developing countries. *Lancet* 2006; 367: 1880-1882.

2. Klugman KP, Madhi SA, Huebner RE, Kohberger R, Mbelle N, Pierce N. A trial of a 9-valent pneumococcal conjugate vaccine in children with and those without HIV infection. *N Engl J Med* 2003; 349: 1341-1348.

3. Collins AM, Wright AD, Mitsi E, Gritzfeld JF, Hancock CA, Pennington SH, Wang D, Morton B, Ferreira DM, Gordon SB. First human challenge testing of a pneumococcal vaccine. Double-blind randomized controlled trial. *Am J Respir Crit Care Med* 2015; 192: 853-858.

4. Alecsandru D, Valor L, Sanchez-Ramon S, Gil J, Carbone J, Navarro J, Rodriguez J, Rodriguez-Sainz C, Fernandez-Cruz E. Sublingual therapeutic immunization with a polyvalent bacterial preparation in patients with recurrent respiratory infections: immunomodulatory effect on antigen-specific memory CD4+ T cells and impact on clinical outcome. *Clin Exp Immunol* 2011; 164: 100-107.

5. Cirauqui C, Benito-Villalvilla C, Sanchez-Ramon S, Sirvent S, Diez-Rivero CM, Conejero L, Brandi P, Hernandez-Cillero L, Ochoa JL, Perez-Villamil B, Sancho D, Subiza JL, Palomares O. Human dendritic cells activated with MV130 induce Th1, Th17 and IL-10 responses via RIPK2 and MyD88 signalling pathways. *Eur J Immunol* 2018; 48: 180-193.

6. Guevara-Hoyer K, Saz-Leal P, Diez-Rivero CM, Ochoa-Grullon J, Fernandez-Arquero M, Perez de Diego R, Sanchez-Ramon S. Trained Immunity Based-Vaccines as a Prophylactic Strategy in Common Variable Immunodeficiency. A Proof of Concept Study. *Biomedicines* 2020; 8.

7. Ochoa-Grullon J, Benavente Cuesta C, Gonzalez Fernandez A, Cordero Torres G, Perez Lopez C, Pena Cortijo A, Conejero Hall L, Mateo Morales M, Rodriguez de la Pena A, Diez-Rivero CM, Rodriguez de Frias E, Guevara-Hoyer K, Fernandez-Arquero M, Sanchez-Ramon S. Trained Immunity-Based Vaccine in B Cell Hematological Malignancies With Recurrent Infections: A New Therapeutic Approach. *Front Immunol* 2020; 11: 611566.

8. Del Fresno C, Garcia-Arriaza J, Martinez-Cano S, Heras-Murillo I, Jarit-Cabanillas A, Amores-Iniesta J, Brandi P, Dunphy G, Suay-Corredera C, Pricolo MR, Vicente N, Lopez-Perrote A, Cabezudo S, Gonzalez-Corpas A, Llorca O, Alegre-Cebollada J, Garaigorta U, Gastaminza P, Esteban M, Sancho D. The Bacterial Mucosal Immunotherapy MV130 Protects Against SARS-CoV-2 Infection and Improves COVID-19 Vaccines Immunogenicity. *Front Immunol* 2021; 12: 748103.

9. Nieto A, Mazon A, Nieto M, Calderon R, Calaforra S, Selva B, Uixera S, Palao MJ, Brandi P, Conejero L, Saz-Leal P, Fernandez-Perez C, Sancho D, Subiza JL, Casanovas M. Bacterial Mucosal Immunotherapy with MV130 Prevents Recurrent Wheezing in Children: A Randomized, Double-Blind, Placebo-controlled Clinical Trial. *Am J Respir Crit Care Med* 2021; 204: 462-472.

10. Brandi P, Conejero L, Cueto FJ, Martinez-Cano S, Dunphy G, Gomez MJ, Relano C, Saz-Leal P, Enamorado M, Quintas A, Dopazo A, Amores-Iniesta J, Del Fresno C, Nistal-Villan E, Ardavin C, Nieto A, Casanovas M, Subiza JL, Sancho D. Trained immunity induction by the inactivated mucosal vaccine MV130 protects against experimental viral respiratory infections. *Cell Rep* 2022; 38: 110184.

11. SanJoaquin MA, Allain TJ, Molyneux ME, Benjamin L, Everett DB, Gadabu O, Rothe C, Nguipdop P, Chilombe M, Kazembe L, Sakala S, Gonani A, Heyderman RS. Surveillance Programme of IN-patients and Epidemiology (SPINE): implementation of an electronic data collection tool within a large hospital in Malawi. *PLoS Med* 2013; 10: e1001400.

12. Swarthout TD, Fronterre C, Lourenco J, Obolski U, Gori A, Bar-Zeev N, Everett D, Kamng'ona AW, Mwalukomo TS, Mataya AA, Mwansambo C, Banda M, Gupta S, Diggle P, French N, Heyderman RS. High residual carriage of vaccine-serotype Streptococcus pneumoniae after introduction of pneumococcal conjugate vaccine in Malawi. *Nat Commun* 2020; 11: 2222.

13. Swarthout TD, Ibarz-Pavon A, Kawalazira G, Sinjani G, Chirombo J, Gori A, Chalusa P, Bonomali F, Nyirenda R, Bulla E, Brown C, Msefula J, Banda M, Kachala J, Mwansambo C, Henrion MY, Gordon SB, French N, Heyderman RS. A pragmatic health centre-based evaluation comparing the effectiveness of a PCV13 schedule change from 3+0 to 2+1 in a high pneumococcal carriage and disease burden setting in Malawi: a study protocol. *BMJ Open* 2021; 11: e050312.

14. Delgleize E, Leeuwenkamp O, Theodorou E, Van de Velde N. Cost-effectiveness analysis of routine pneumococcal vaccination in the UK: a comparison of the PHiD-CV vaccine and the PCV-13 vaccine using a Markov model. *BMJ open* 2016; 6: e010776-e010776.

15. Swarthout TF, C; Lourenço, J: Obolski, U; Gori, A; Bar-Zeev, N; Everett, D; Kamngona, AX; Mwalukomo, TS; Mataya, AA; Mwansambo, C; Banda, M; Gupta, S; Diggle, P; French, N; Heyderman, RS. High residual prevalence of vaccine serotype Streptococcus pneumoniae carriage 4 to 6 years after introduction of 13-valent pneumococcal conjugate vaccine in Malawi: a prospective serial cross-sectional study. 2018 08/01/2019]. Available from: <https://doi.org/10.1101/445999>

16. Morton B, Burr S, Chikaonda T, Nsomba E, Manda-Taylor L, Henrion MYR, Banda NP, Rylance J, Ferreira DM, Jambo K, Gordon SB, consortium M. A feasibility study of controlled human infection with Streptococcus pneumoniae in Malawi. *EBioMedicine* 2021; 72: 103579.

17. Morton B, Jambo K, Chikaonda T, Rylance J, Henrion MYR, Banda NP, Nsomba E, Gondwe J, Ferreira D, Gordon SB, Consortium M. The influence of pneumococcal conjugate vaccine-13 on nasal colonisation in a controlled human infection model of pneumococcal carriage in Malawi: a double-blinded randomised controlled trial protocol. *Wellcome Open Res* 2021; 6: 240.

18. Roukens AHE, Pothast CR, Konig M, Huisman W, Dalebout T, Tak T, Azimi S, Kruize Y, Hagedoorn RS, Zlei M, Staal FJT, de Bie FJ, van Dongen JJM, Arbous SM, Zhang JLH, Verheij M, Prins C, van der Does AM, Hiemstra PS, de Vries JJC, Janse JJ, Roestenberg M, Myeni SK, Kikkert M, Yazdanbakhsh M, Heemskerk MHM, Smits HH, Jochems SP, in collaboration with B-Cg, in collaboration with C-Lg. Prolonged activation of nasal immune cell populations and development of tissue-resident SARS-CoV-2-specific CD8(+) T cell responses following COVID-19. *Nature immunology* 2022; 23: 23-32.

19. Mtunthama Toto N, Gooding K, Kapumba BM, Jambo K, Rylance J, Burr S, Morton B, Gordon SB, Manda-Taylor L. "At first, I was very afraid"-a qualitative description of participants' views and experiences in the first Human Infection Study in Malawi. *Wellcome Open Res* 2021; 6: 89.

20. Kapumba BM, Jambo K, Rylance J, Gmeiner M, Sambakunsi R, Parker M, Gordon SB, Gooding K. Stakeholder views on the acceptability of human infection studies in Malawi. *BMC Med Ethics* 2020; 21: 14.

21. Garcia Gonzalez LA, Arrutia Diez F. Mucosal bacterial immunotherapy with MV130 highly reduces the need of tonsillectomy in adults with recurrent tonsillitis. *Hum Vaccin Immunother* 2019; 15: 2150-2153.

22. Sanchez-Ramon S, Fernandez-Paredes L, Saz-Leal P, Diez-Rivero CM, Ochoa-Grullon J, Morado C, Macarron P, Martinez C, Villaverde V, de la Pena AR, Conejero L, Hernandez-Llano K, Cordero G, Fernandez-Arquero M, Gutierrez BF, Candelas G. Sublingual Bacterial Vaccination Reduces Recurrent Infections in Patients With Autoimmune Diseases Under Immunosuppressant Treatment. *Front Immunol* 2021; 12: 675735.

23. Naclerio RM, Meier HL, Kagey-Sobotka A, Adkinson NF, Jr., Meyers DA, Norman PS, Lichtenstein LM. Mediator release after nasal airway challenge with allergen. *Am Rev Respir Dis* 1983; 128: 597-602.

24. Ferreira DM, Neill DR, Bangert M, Gritzfeld JF, Green N, Wright AK, Pennington SH, Bricio-Moreno L, Moreno AT, Miyaji EN, Wright AD, Collins AM, Goldblatt D, Kadioglu A, Gordon SB. Controlled human infection and rechallenge with Streptococcus pneumoniae reveals the protective efficacy of carriage in healthy adults. *Am J Respir Crit Care Med* 2013; 187: 855-864.

25. Morton B, Burr S, Jambo K, Rylance J, Henrion MYR, Banda NP, Nsomba E, Kapumba B, Manda-Taylor L, Masesa C, Ferrreira D, Gordon SB, Consortium M. A pneumococcal controlled human infection model in Malawi: Transfer of an established pneumococcal carriage model from Liverpool, UK to Blantyre, Malawi - A feasibility study. *Wellcome Open Res* 2020; 5: 25.

26. Auranen K, Rinta-Kokko H, Goldblatt D, Nohynek H, O'Brien KL, Satzke C, Simell B, Tanskanen A, Kayhty H. Colonisation endpoints in Streptococcus pneumoniae vaccine trials. *Vaccine* 2013; 32: 153-158.

27. Morton B, Burr S, Jambo K, Rylance J, Henrion M, Banda N, Nsomba E, Kapumba B, Manda-Taylor L, Masesa C, Ferrreira D, Gordon S, null n. A pneumococcal controlled human infection model in Malawi: Transfer of an established pneumococcal carriage model from Liverpool, UK to Blantyre, Malawi ? A feasibility study [version 1; peer review: awaiting peer review]. *Wellcome open research* 2020; 5.

28. PHE. <https://www.gov.uk/government/publications/wuhan-novel-coronavirus-infection-prevention-and-control/covid-19-personal-protective-equipment-ppe>. 2020 23/06/2020]. Available from: <https://www.gov.uk/government/publications/wuhan-novel-coronavirus-infection-prevention-and-control/covid-19-personal-protective-equipment-ppe>.

29. Gordon SB, Chinula L, Chilima B, Mwapasa V, Dadabhai S, Mlomb Y. A Malawi guideline for research study participant remuneration. 2018 09/12/2018]. Available from: <https://wellcomeopenresearch.org/articles/3-141/v1>.

30. Dherani MK, Pope D, Tafatatha T, Heinsbroek E, Chartier R, Mwalukomo T, Crampin A, Mitsi E, German EL, Nikolaou E, Solorzano C, Ferreira DM, Swarthout TD, Hinds J, Mortimer K, Gordon SB, French N, Bruce NG. Association between household air pollution and nasopharyngeal pneumococcal carriage in Malawian infants (MSCAPE): a nested, prospective, observational study. *Lancet Glob Health* 2022; 10: e246-e256.

31. Abruzzo AR, Aggarwal SD, Sharp ME, Bee GCW, Weiser JN. Serotype-Dependent Effects on the Dynamics of Pneumococcal Colonization and Implications for Transmission. *mBio* 2022; 13: e0015822.
