# Supplementary material for: Whole cell inactivated poly-bacterial preparation MV130 effect on nasal mucosal immunity and experimental human pneumococcal carriage: double-blind randomised controlled trial with controlled human infection model
Source: eBioMedicine. 2026 Aug 26;131:106443. doi: 10.1016/j.ebiom.2026.106443 (PMC13544312; doi:10.1016/j.ebiom.2026.106443)
Supplement: Supplementary file 4 [file mmc4.docx]

**MARVELS CONSORTIUM MEMBERS**

**MARVELS consortium members:** Mark Alderson^1^, Sandra Antoine^2^, Christopher R Bailey^3^, Debbie Bogaert^4^, Jeremy Brown^5^, Sarah Burr^6^, Marien L De Jonge^7^, Klara Doherty^2,8^, David Goldblatt^5^, Gabriela Gomes^9^, Joel Gondwe^2^, Kate Gooding^1^, Tina Harawa^2^, Rob Heyderman^5^, Jason Hinds^10^, Simon Jochems^11^, Blessings Kapumba^2^, Vella Kaudzu^2^, Robert Kneller^13^, Richard Malley^14^, Jane Mallewa^15^, Lucinda Manda-Taylor^15^, Henry Mwandumba^2^, Percy Mwenechanya^2^, Mike Parker^16^, Andrew Pollard^16^, Modesta Reuben^15^, and Jeffrey Weiser^17^.

1. PATH, USA
2. Malawi-Liverpool-Wellcome Research Programme, Blantyre, Malawi
3. ImmBio, UK
4. The University of Edinburgh, UK
5. University College London, UK
6. Liverpool School of tropical medicine
7. Radboud University Medical Center, Netherlands
8. Department of Clinical Sciences, Liverpool School of Tropical Medicine, Liverpool, UK
9. University of Srathclyde
11. St Georges, University London, UK
12. Leiden University medical center
13. University of Tokyo, Japan
14. Boston Children’s Hospital, USA
15. Kamuzu University of Health Sciences, Malawi
16. University of Oxford
17. New York University, Grossman School of Medicine, USA
